# PCSK9 Inhibition Attenuates Renal Oxidative Stress and Lipotoxicity in Diabetic Kidney Disease in Association with AMPK Activation

**DOI:** 10.3390/antiox15091182

**Published:** 2026-09-17

**Authors:** Keum-Jin Yang, Sunha Lee, Sungmi Kim, Sojung Youn, Hwajin Park, Yunkyeong Hwang, Suyeon Han, Yoon-Kyung Chang, Cheol Whee Park, Yu Ah Hong

**Affiliations:** 1 Clinical Research Institute, Daejeon St. Mary’s Hospital, Daejeon 34943, Republic of Korea; nadia@cnu.ac.kr (K.-J.Y.);; 2 Division of Nephrology, Department of Internal Medicine, Eunpyeong St. Mary’s Hospital, College of Medicine, The Catholic University of Korea, Seoul 06591, Republic of Korea; 3 Division of Nephrology, Department of Internal Medicine, Bucheon St. Mary’s Hospital, College of Medicine, The Catholic University of Korea, Seoul 06591, Republic of Korea; 4 Division of Nephrology, Department of Internal Medicine, Daejeon St. Mary’s Hospital, College of Medicine, The Catholic University of Korea, Seoul 06591, Republic of Korea; 5 Division of Nephrology, Department of Internal Medicine, Seoul St. Mary’s Hospital, College of Medicine, The Catholic University of Korea, Seoul 06591, Republic of Korea

**Keywords:** diabetic nephropathies, proprotein convertase subtilisin/kexin type 9, AMP-activated protein kinase, oxidative stress, apoptosis

## Abstract

Renal lipotoxicity and oxidative stress are major contributors to diabetic kidney disease (DKD), but the role of proprotein convertase subtilisin/kexin type 9 (PCSK9) and its relationship with AMP-activated protein kinase (AMPK) signaling remain unclear. We investigated the effects of PCSK9 inhibition on renal lipotoxicity and oxidative stress in *db*/*db* mice and high-glucose (HG)-treated HK-2 cells using a selective PCSK9 inhibitor (PCSK9i) with or without *AMPKα1*/*α2* small interfering RNA (siRNA). PCSK9i significantly reduced albuminuria, renal injury, histopathological changes, and intrarenal lipid accumulation without altering glycemic control. These effects were accompanied by increased low-density lipoprotein receptor (LDLR) expression, reduced cluster of differentiation 36 (CD36) expression, enhanced AMPK phosphorylation and peroxisome proliferator-activated receptor gamma coactivator-1α (PGC-1α) expression, reduced sterol regulatory element-binding protein-1c (SREBP-1c) expression, restored carnitine palmitoyltransferase 1 (CPT1) expression, modulated protein kinase B (Akt)/forkhead box O (FoxO) signaling, and reduced oxidative stress and apoptosis. Similar findings were observed in HG-treated HK-2 cells. Importantly, AMPK knockdown attenuated the protective effects of PCSK9i on lipid metabolism, oxidative stress, and apoptosis in HK-2 cells. PCSK9 inhibition attenuates renal lipotoxicity and oxidative stress in DKD, providing a preclinical rationale for further investigation of the renal metabolic effects of PCSK9-directed therapies and their association with AMPK activation.

## 1. Introduction

Diabetic kidney disease (DKD), the leading cause of end-stage kidney disease (ESKD) worldwide, remains a major contributor to cardiovascular morbidity and mortality. In South Korea, approximately half of patients receiving dialysis have DKD as the primary cause of kidney failure [1]. Although recent advances in glucose-lowering and kidney-protective therapies have improved clinical outcomes, DKD progression remains driven by mechanisms beyond hyperglycemia, including renal hypoxia, metabolic dysregulation, chronic inflammation, and mitochondrial dysfunction [2]. In addition to glucotoxicity, lipotoxicity, which is characterized by ectopic lipid accumulation and lipid-induced cellular stress, has emerged as a pivotal driver of renal injury [3]. Because the kidney is a highly energy-demanding organ, disruption of lipid homeostasis profoundly impairs cellular metabolism and promotes progressive renal damage.

AMP-activated protein kinase (AMPK) is a central regulator of cellular energy metabolism that is activated during metabolic stress. Upon activation, AMPK promotes catabolic pathways while inhibiting anabolic processes, thereby restoring energy homeostasis [4,5]. Beyond its energy-sensing role, AMPK regulates fatty acid oxidation, glucose uptake, glycolysis, autophagy, and mitochondrial biogenesis and enhances cellular antioxidant defenses [5,6]. Experimental studies have demonstrated that impaired AMPK signaling contributes to renal lipid accumulation and accelerates DKD progression, whereas pharmacological activation of AMPK ameliorates renal lipotoxicity and kidney injury [7,8,9]. Despite the well-established protective effects of AMPK activation, the upstream mechanisms responsible for AMPK dysregulation in DKD remain incompletely understood.

Proprotein convertase subtilisin/kexin type 9 (PCSK9), a serine protease of the proteinase K subfamily primarily involved in lipid metabolism, promotes lysosomal degradation of low-density lipoprotein receptor (LDLR) [10]. However, accumulating evidence indicates that PCSK9 also exerts tissue-specific metabolic effects beyond hepatic lipid regulation. PCSK9 is expressed in extrahepatic tissues, including the kidney, where it is predominantly localized in proximal tubular epithelial cells [11]. Elevated plasma PCSK9 concentrations have been associated with nephrotic syndrome [12] and type 2 DKD [13]. Renal PCSK9 expression also correlates with the severity of disease in experimental models of nephrotic syndrome [14,15]. However, whether PCSK9 regulates renal lipid metabolism through AMPK signaling and thereby contributes to DKD progression remains unknown.

Given the central role of AMPK in lipid metabolism, PCSK9 may contribute to DKD progression through modulation of AMPK signaling. This study aimed to determine whether PCSK9 inhibition modulates AMPK and attenuates renal lipotoxicity in DKD. We hypothesized that PCSK9 inhibition attenuates lipid accumulation-induced oxidative stress and apoptosis by activating AMPK signaling in DKD.

## 2. Materials and Methods

### 2.1. Animal Experiments

Eight-week-old male C57BLKS/J *db*/*m* control and *db*/*db* mice (Jackson Laboratory, Bar Harbor, ME, USA) were assigned within each genotype before treatment initiation to the vehicle- or PCSK9i-treated group to achieve comparable baseline body weights between the treatment groups, resulting in four experimental groups: *db*/*m* control (*n* = 6), *db*/*m* + PCSK9 inhibitor (PCSK9i) (*n* = 6), *db*/*db* control (*n* = 8), and *db*/*db* + PCSK9i (*n* = 8). Mice in the PCSK9i groups received SBC-115076 (1.5 mg/kg/day; Sigma-Aldrich, St. Louis, MO, USA), a selective PCSK9 inhibitor, via subcutaneous injection once daily for 8 weeks. SBC-115076 was dissolved in 0.5% carboxymethyl cellulose, and control groups received an equivalent volume of vehicle. Body weight and fasting blood glucose levels were monitored weekly throughout the treatment period, and the final measurements were used for statistical analysis.

At the end of the treatment, mice were fasted for 8 h and euthanized by CO_2_ inhalation, followed by cervical dislocation. Blood, urine, kidney, and liver tissues were subsequently collected. All animal procedures were approved by the Institutional Animal Care and Use Committee of Daejeon St. Mary’s Hospital (CMCDJ-AP-2020-008) and performed in accordance with institutional guidelines.

### 2.2. Biochemical Analysis

Fasting blood glucose levels were measured using an Accu-Chek meter (Roche Diagnostics, Indianapolis, IN, USA), and HbA1c levels were analyzed with a DCA Vantage Analyzer (Siemens Healthineers, Erlangen, Germany). Serum insulin and cystatin C concentrations were quantified using ELISA kits. The homeostatic model assessment for insulin resistance (HOMA-IR) was calculated as follows: fasting insulin (μU/mL) × fasting glucose (mg/dL)/405. Serum and intrarenal triglyceride (TG), urinary creatinine, and serum aspartate aminotransferase (AST) and alanine aminotransferase (ALT) levels were measured using the IDEXX VetTest^®^ Chemistry Analyzer (IDEXX Laboratories, Inc., Westbrook, ME, USA). Serum low-density lipoprotein cholesterol (LDL-C) and non-esterified fatty acid (NEFA) levels, as well as urinary albumin concentrations, were quantified using colorimetric assay kits. Urinary albumin excretion was assessed using the albumin-to-creatinine ratio (ACR). Serum and urinary kidney injury molecule-1 (KIM-1) and neutrophil gelatinase-associated lipocalin (NGAL) levels were quantified using ELISA kits. Urinary KIM-1 and NGAL levels were normalized to urinary creatinine levels. Assay details are summarized in Supplementary Table S1.

### 2.3. Histological and Immunohistochemical Analysis

Paraffin-embedded kidney sections were stained with periodic acid–Schiff (PAS) to assess glomerular mesangial expansion. The mesangial matrix area was quantified in ≥20 glomeruli per kidney and expressed as a percentage of the total glomerular area. For immunohistochemistry, 4 µm kidney sections were incubated overnight at 4 °C with primary antibodies against transforming growth factor-β1 (TGF-β1), type IV collagen (Col IV), 8-hydroxy-2′-deoxyguanosine (8-OHdG) and PCSK9. Glomerular TGF-β1-positive cells were counted and expressed per high-power field (HPF), whereas glomerular Col IV staining and glomerular and tubular 8-OHdG staining were quantified as percentages of positively stained area in ≥20 fields per slide. PCSK9 staining was scored from 0 to 12 based on intensity (0−4) and extent (0−3) [16]. Apoptotic cells were detected using the terminal deoxynucleotidyl transferase dUTP nick-end labeling (TUNEL) assay and TUNEL-positive cells were quantified by cell counting. ImageJ software Version 1.54p (National Institutes of Health, Bethesda, MD, USA; https://imagej.net/ij/; accessed on 7 September 2026) was used for area-based image analysis. Histological and immunohistochemical assessments were performed by investigators blinded to group allocation. Detailed information is provided in Supplementary Tables S1 and S2.

### 2.4. In Vitro Experiments

Human proximal tubular epithelial cells (HK-2; American Type Culture Collection [ATCC], Manassas, VA, USA; passages 6–15) were maintained in DMEM supplemented with 10% FBS and 1% penicillin–streptomycin (50 U/mL and 50 μg/mL, respectively) at 37 °C in a humidified atmosphere containing 5% CO_2_. Cell line identity was authenticated by ATCC using short tandem repeat profiling before distribution; no additional in-house STR profiling was performed. Cells were tested for mycoplasma contamination using a Mycoplasma PCR Detection Kit (Abcam, Cambridge, UK) and confirmed to be mycoplasma-negative. Cells were assigned to either a low-glucose (LG) control (5.6 mM D-glucose plus 27.4 mM D-mannitol as an osmolality balance) or a high-glucose (HG) group (33 mM D-glucose). At 60–70% confluence, cells were pretreated with 0.25 μM SBC-115076 or an equivalent volume of vehicle for 1 h and subsequently exposed to HG for 48 h. The concentration and exposure duration of SBC-115076 were selected based on preliminary optimization experiments. Cell viability was assessed using an MTT assay, and treatment with 0.25 μM SBC-115076 for 48 h did not significantly affect HK-2 cell viability compared with vehicle treatment (Supplementary Figure S1).

To investigate the functional involvement of AMPK in the effects of PCSK9 inhibition under HG conditions, transient gene silencing was performed. HK-2 cells at 60–70% confluence were transfected for 24 h with siRNA targeting either human *PRKAA1* (25 nM) or *PRKAA2* (50 nM), or with scrambled control siRNA (Supplementary Table S3) using Lipofectamine RNAiMAX (Thermo Fisher Scientific, Waltham, MA, USA) in Opti-MEM medium (Gibco, Grand Island, NY, USA). We confirmed significant knockdown of AMPKα1 and AMPKα2 beginning at 24 h after transfection through time-course analyses of isoform-specific protein expression (Supplementary Figure S2). For the functional experiments, cells were transfected with siRNA for 24 h, pretreated with SBC-115076 for 1 h, and subsequently exposed to HG for 48 h before harvesting and analysis. All in vitro experiments were independently repeated at least three times.

### 2.5. Real-Time Reverse Transcription Polymerase Chain Reaction

Total RNA was extracted from kidney tissues using TRIzol reagent (Invitrogen, Carlsbad, CA, USA) and complementary DNA was synthesized using a Reverse Transcriptase Premix Kit (Elpis Biotech, Daejeon, Republic of Korea). Quantitative real-time polymerase chain reaction (qRT-PCR) was performed with Power SYBR^®^ Green PCR Master Mix on the ABI 7500 Fast System (Applied Biosystems, Foster City, CA, USA). Gene-specific primers were used to quantify mRNA levels of PCSK9, sterol regulatory element-binding protein-1c (SREBP-1c), perilipin, peroxisome proliferator–activated receptor α (PPARα), peroxisome proliferator–activated receptor γ (PPARγ), and carnitine palmitoyltransferase 1 (CPT1). Glyceraldehyde 3-phosphate dehydrogenase (GAPDH) was used as an internal control, and gene expression levels were analyzed using the 2^−ΔΔCt^ method. All qRT-PCR reactions were performed in triplicate. Primer sequences are listed in Supplementary Table S4.

### 2.6. Immunoblot Analysis

Proteins extracted from kidney tissues and HK-2 cells were quantified using a bicinchoninic acid protein assay (Thermo Fisher Scientific, Waltham, MA, USA) and separated using sodium dodecyl sulfate-polyacrylamide gel electrophoresis, followed by transfer to nitrocellulose membranes. Membranes were incubated with primary antibodies against the following proteins: PCSK9, LDLR, cluster of differentiation 36 (CD36), total AMPK, phospho-Thr^172^ AMPK, peroxisome proliferator-activated receptor-γ coactivator (PGC)-1α, total Akt, phospho-Ser^473^ Akt, total forkhead box O transcription factor (FoxO)1, phospho-Ser^256^ FoxO1, total FoxO3a, phospho-Ser^253^ FoxO3a, SREBP-1c, SREBP2, Perilipin, CPT1, PPARα, PPARγ, Bcl-2 associated X (Bax), and B-cell lymphoma 2 (Bcl-2). Immunoreactive bands were visualized using a chemiluminescent detection system (Amersham Pharmacia Biotech, Little Chalfont, UK) and the ChemiDoc™ XRS+ imaging system (Bio-Rad Laboratories, Hercules, CA, USA). GAPDH and β-actin were used as loading controls. Antibody details are provided in Supplementary Table S2.

### 2.7. Assessment of Lipid Metabolism and Oxidative Stress

Intrarenal lipids were extracted using a modified Bligh and Dyer method [17]. Levels of intrarenal LDL-C and NEFA were quantified using commercial assay kits. Kidney TG levels were determined using the same method as for serum. In HK-2 cells, lipid accumulation was evaluated using BODIPY staining. Cells were stained with BODIPY, washed with PBS, and mounted with Vectashield Antifade Mounting Medium containing DAPI (Vector Laboratories, Newark, CA, USA). Lipid droplets were visualized using bright-field and confocal microscopy (LSM880 with Airyscan; Carl Zeiss, Oberkochen, Germany) and quantified using ImageJ.

Lipid peroxidation was evaluated by measuring malondialdehyde (MDA) levels in kidney tissues and HK-2 cells. Long-chain fatty acid oxidation (FAO-L) activity was measured in HK-2 cell lysates using a palmitoyl-CoA-based colorimetric assay. After incubation at 37 °C for 60 min, absorbance was measured at 492 nm using a microplate reader (Bio-Rad Laboratories, Hercules, CA, USA). The corresponding control-well value was subtracted, and FAO-L activity was normalized to total protein concentration. Detailed assay information is provided in Supplementary Table S1.

### 2.8. Measurement of Oxidative Stress and Apoptosis in HK-2 Cells

To assess the antioxidant and anti-apoptotic effects of PCSK9i in HK-2 cells, intracellular oxidative stress was measured using 2′,7′-dichlorofluorescein diacetate (DCF-DA), a nonfluorescent, cell-permeable probe. After the indicated treatments, HK-2 cells were incubated with 10 μM DCF-DA in the presence or absence of PCSK9i for 30 min at 37 °C. Following incubation, cells were washed with phosphate-buffered saline, and fluorescence was detected using a fluorescence microscope (Eclipse TE300, Nikon, Tokyo, Japan). Quantification of DCF-derived fluorescence intensity was performed with ImageJ. Apoptosis was assessed using a TUNEL assay according to the manufacturer’s instructions. TUNEL-positive cells were counted using ImageJ and expressed as a percentage of total cells. Detailed assay information is provided in Supplementary Table S1.

### 2.9. Statistical Analysis

Data are presented as mean ± standard deviation (SD). For the in vivo analyses, the normality of model residuals was assessed using the Shapiro–Wilk test, and homogeneity of variance was assessed using Levene’s test. Outcomes from complete 2 × 2 factorial experiments were analyzed using two-way analysis of variance (ANOVA), followed by Tukey’s multiple-comparisons test. Five-group experiments involving *PRKAA1* or *PRKAA2* siRNAs were analyzed using one-way ANOVA followed by Tukey’s multiple-comparisons test. Statistical analyses were performed using SPSS Statistics (version 20.0; IBM Corp., Armonk, NY, USA) and GraphPad Prism (version 9.5.1; GraphPad Software, Boston, MA, USA). A *p* value < 0.05 was considered statistically significant, and Tukey-adjusted *p* values are reported for post hoc pairwise comparisons.

## 3. Results

### 3.1. PCSK9 Inhibition Reduces Albuminuria, Liver Enzymes, and Lipid Profiles in Diabetic Mice

**Figure 1:**
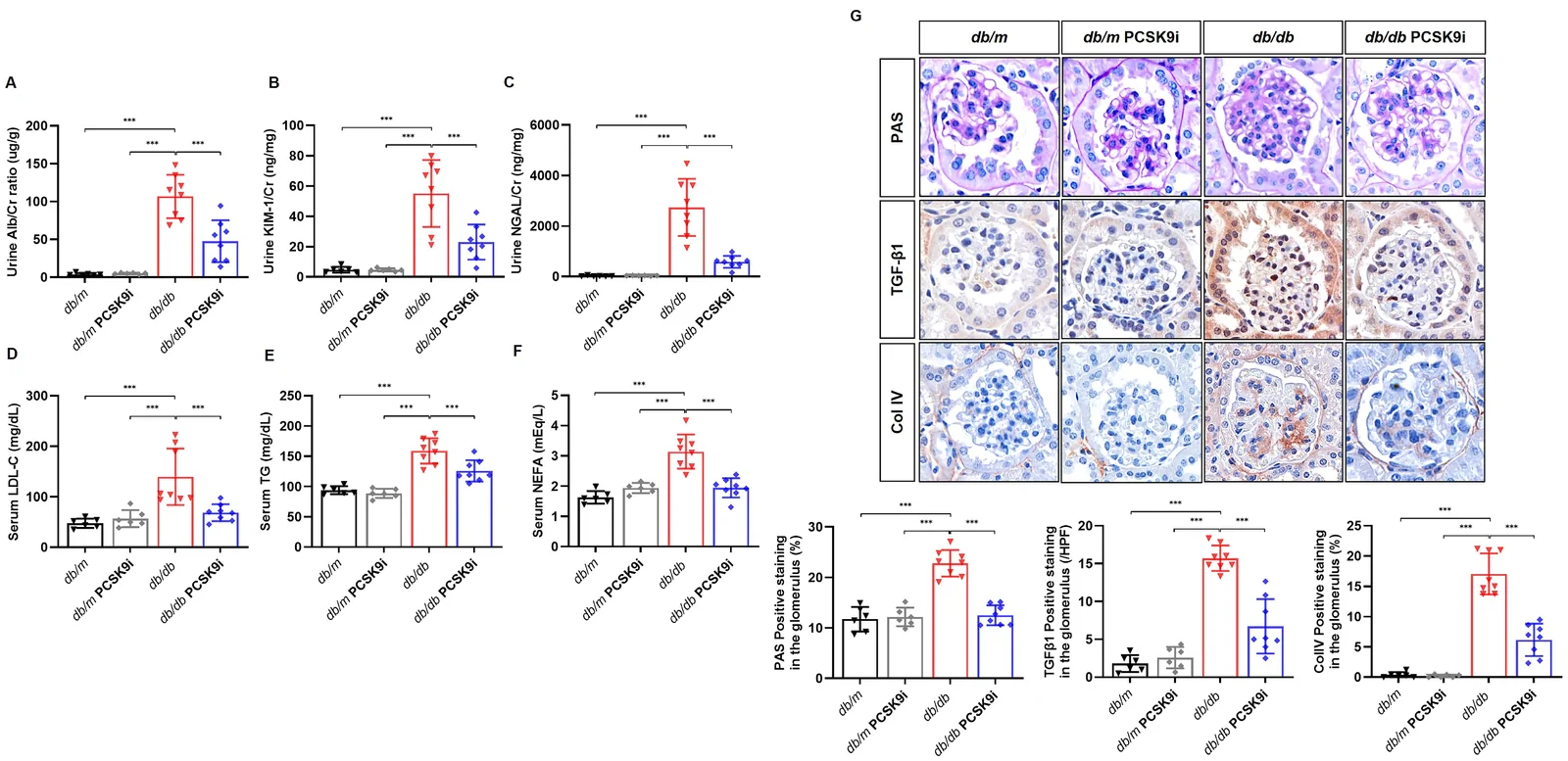
Effects of PCSK9 inhibition on urinary kidney injury markers, lipid profiles, and renal histologic alterations. (**A**) Urinary albumin/creatinine ratio (ACR), (**B**) urinary kidney injury molecule-1 (KIM-1)/creatinine ratio, and (**C**) urinary neutrophil gelatinase-associated lipocalin (NGAL)/creatinine ratio were significantly increased in *db*/*db* mice and attenuated by PCSK9i treatment. (**D**–**F**) Serum lipid profiles, including low-density lipoprotein cholesterol (LDL-C), triglycerides (TG), and non-esterified fatty acids (NEFA), were elevated in *db*/*db* mice and reduced by PCSK9i. (**G**) Representative kidney sections stained with periodic acid–Schiff (PAS), and immunohistochemical staining for transforming growth factor-β1 (TGF-β1) and type IV collagen (Col IV) in the glomerulus are shown (original magnification, ×400). Quantitative analysis of mesangial fractional area, TGF-β1-positive cells, and Col IV-positive area is also presented. Data are expressed as mean ± SD (*n* = 6 per group: *db*/*m* and *db*/*m* + PCSK9i, *n* = 8 per group *db*/*db* and *db*/*db* + PCSK9i). Tukey-adjusted *p* for the indicated comparisons: *** *p* < 0.001.

PCSK9i treatment did not alter body weight, fasting blood glucose levels, HbA1c, HOMA-IR or serum cystatin C levels in either *db*/*m* or *db*/*db* mice (all adjusted *p* > 0.05). Diabetic mice showed increased AST and ALT levels (*p* = 0.013 and *p* < 0.001, respectively), which were significantly reduced following PCSK9i, indicating that the observed effects were independent of glycemic control (all *p* < 0.001; Table 1). Urinary ACR was increased in *db*/*db* mice and reduced by PCSK9i treatment by a mean difference of 77.7 μg/mg Cr (95% CI, 51.1–104; *p* < 0.001; Figure 1A). Serum KIM-1 and NGAL levels and urinary KIM-1/Cr and NGAL/Cr ratios were similarly increased in *db*/*db* mice and reduced by PCSK9i treatment (all *p* < 0.001; Table 1, Figure 1B,C). Serum LDL-C, TG, and NEFA levels were elevated in *db*/*db* mice but were significantly reduced by PCSK9i treatment (all *p* < 0.001; Figure 1D–F). The glomerular mesangial fractional area was increased in *db*/*db* mice and reduced by PCSK9i treatment by a mean difference of 10 percentage points (95% CI, 7.2–13; *p* < 0.001; Figure 1G). TGF-β1 and Col IV expression were similarly increased in *db*/*db* mice and reduced by PCSK9i treatment (both *p* < 0.001; Figure 1G). In addition, excessive hepatic lipid droplet accumulation observed in *db*/*db* mice was significantly reduced by PCSK9i (Supplementary Figure S3).

### 3.2. PCSK9 Inhibition Reduces Intrarenal Lipids and Modulates LDLR and CD36 in Diabetic Mice

**Figure 2:**
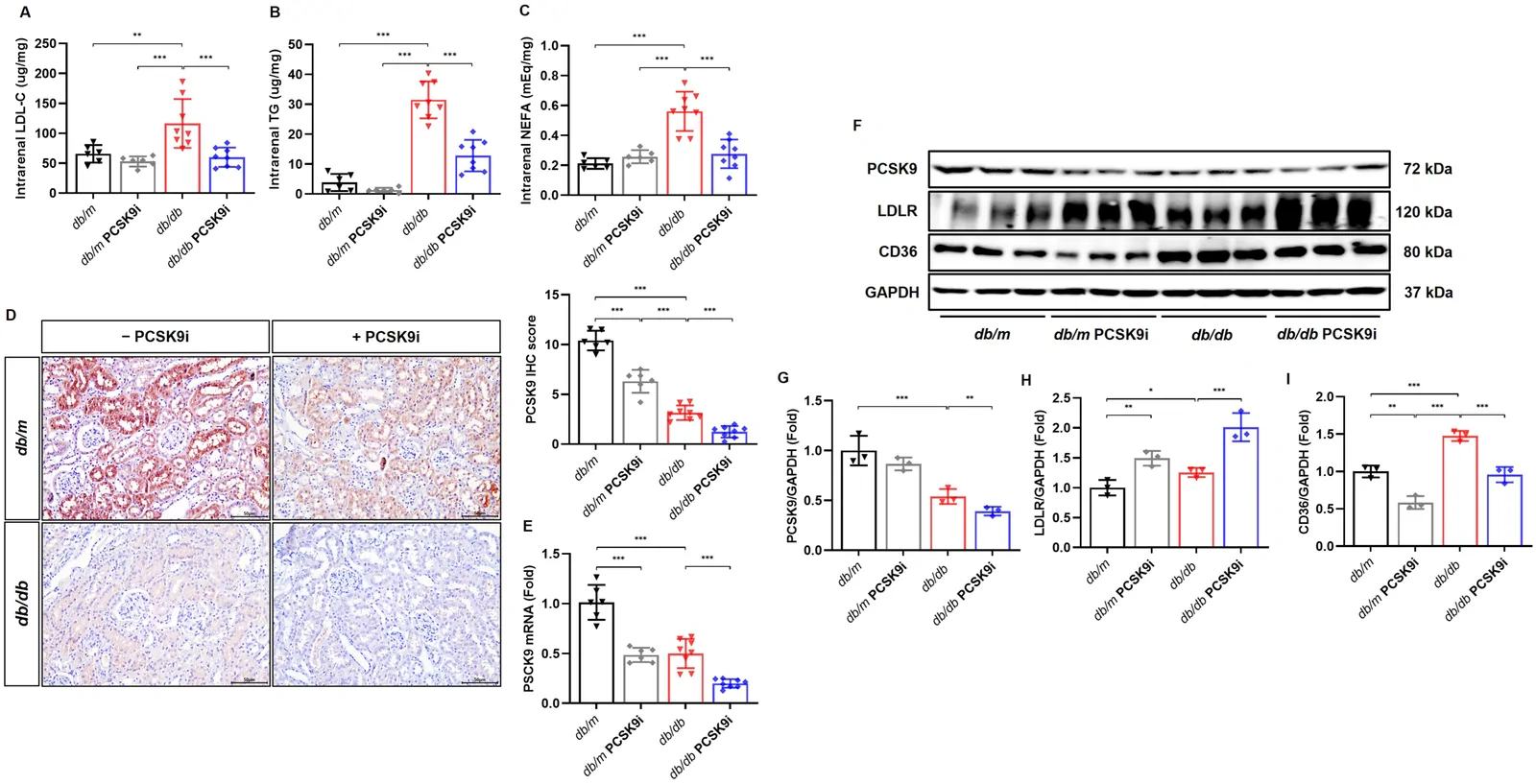
PCSK9 inhibition reduces intrarenal lipid levels and affects renal expression of PCSK9, LDLR, and CD36. (**A**–**C**) Intrarenal levels of low-density lipoprotein cholesterol (LDL-C), triglycerides (TG), and non-esterified fatty acids (NEFA) were significantly elevated in *db*/*db* mice and reduced following PCSK9i treatment. (**D**) Representative immunohistochemical staining of PCSK9. PCSK9 expression was markedly decreased, particularly in the proximal tubules of *db*/*db* mice and further reduced by PCSK9i (original magnification ×200). (**E**) mRNA expression levels of PCSK9 in kidney tissues were significantly lower in *db*/*db* mice than in *db*/*m* controls and further decreased following PCSK9i treatment. (**F**) Representative immunoblots for the expression of PCSK9, low-density lipoprotein receptor (LDLR), and cluster of differentiation 36 (CD36). (**G**–**I**) Quantitative analysis of PCSK9, LDLR, and CD36 protein expression normalized to β-actin. PCSK9i treatment further enhanced LDLR expression and reduced CD36 expression in *db*/*db* mice. Data are expressed as mean ± SD (PCSK9 immunohistochemistry and mRNA analysis, *n* = 6 per group: *db*/*m* and *db*/*m* + PCSK9i, *n* = 8 per group *db*/*db* and *db*/*db* + PCSK9i; for immunoblot analyses, *n* = 3 per group). Tukey-adjusted *p* for the indicated comparisons: * *p* < 0.05, ** *p* < 0.01, and *** *p* < 0.001.

To assess intrarenal lipid accumulation, intrarenal LDL-C, TG, and NEFA levels, as well as the expression of PCSK9, LDLR, and CD36, were examined. Intrarenal NEFA levels were increased in *db*/*db* mice and reduced by PCSK9 inhibition by a mean difference of 0.28 mEq/mg (95% CI, 0.16–0.41; *p* < 0.001; Figure 2C). Intrarenal LDL-C levels were increased in *db*/*db* mice (*p* = 0.005) and reduced by PCSK9 inhibition (*p* < 0.001). Intrarenal TG levels were similarly increased and reduced (both *p* < 0.001; Figure 2A,B). Immunohistochemical analysis demonstrated that PCSK9 was predominantly localized to proximal tubules of the kidney in *db*/*m* control mice, whereas its expression was reduced in *db*/*db* mice and further suppressed by PCSK9i treatment (all *p* < 0.001; Figure 2D). PCSK9 mRNA and protein levels also showed a significant decrease in *db*/*db* mice (both *p* < 0.001) and were further decreased by PCSK9i in *db*/*db* mice (*p* < 0.001 and *p* = 0.004, respectively; Figure 2E–G). LDLR and CD36 expression levels were elevated in *db*/*db* mice (*p* = 0.046 and *p* < 0.001, respectively). PCSK9 inhibition further enhanced LDLR expression while significantly decreasing CD36 expression (both *p* < 0.001; Figure 2F,H,I).

### 3.3. PCSK9 Inhibition Activates AMPK Signaling and Modulates FoxO Pathways in Diabetic Mice

**Figure 3:**
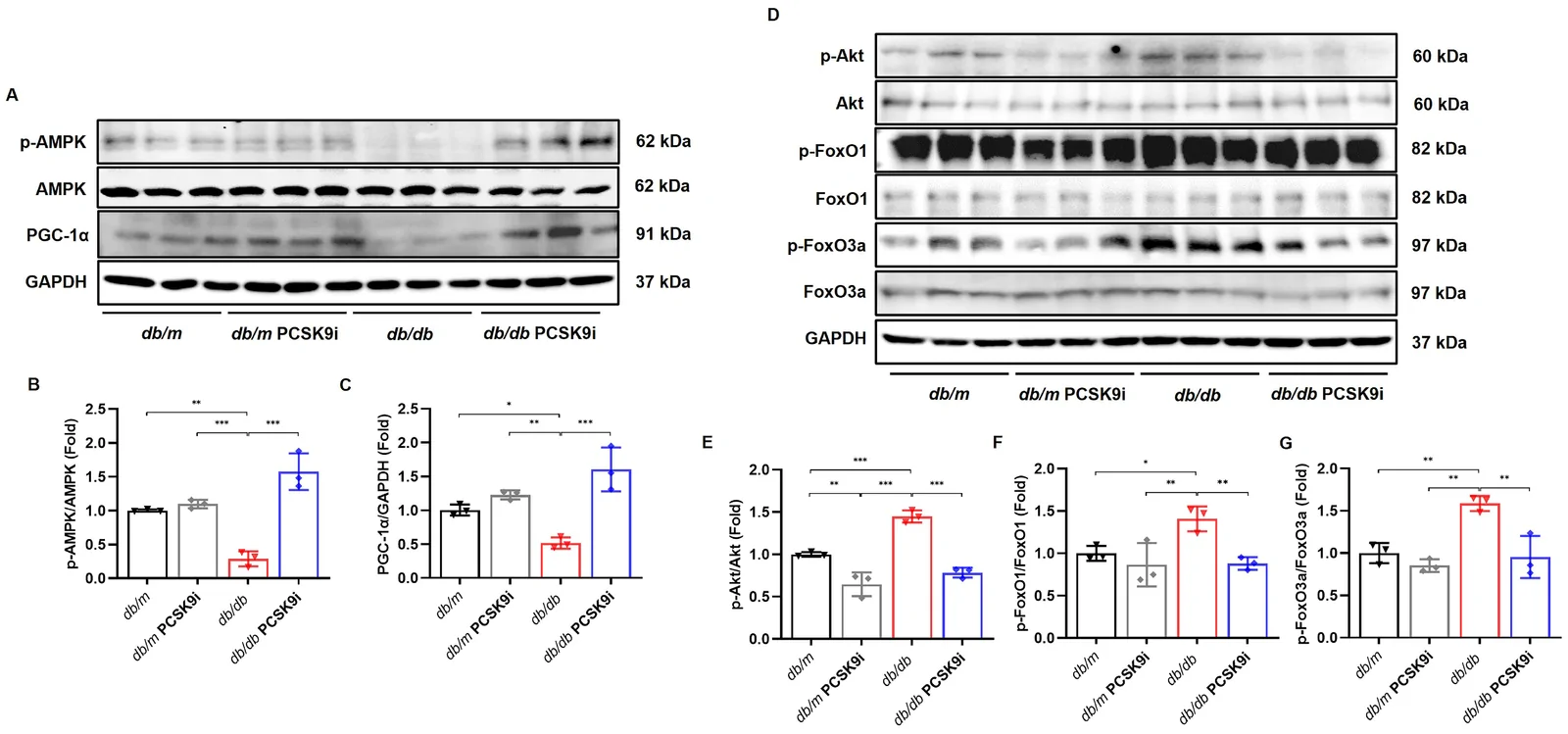
PCSK9 inhibition affects AMPK activation and its downstream signaling pathways in the kidneys. (**A**) Representative immunoblots showing phosphorylated and total AMPK levels and PGC-1α levels in kidney tissues from *db*/*m* and *db*/*db* mice treated with or without PCSK9i. (**B**,**C**) Quantitative analyses of phospho-Thr^172^ AMPK to total AMPK and PGC-1α. (**D**) Representative immunoblots showing phosphorylated and total levels of Akt, FoxO1, and FoxO3a in kidney tissues from *db*/*m* and *db*/*db* mice treated with or without PCSK9i. (**E**–**G**) Quantitative analyses of phospho-Ser^473^ Akt to total Akt, phospho-Ser^256^ FoxO1 to total FoxO1, and phospho-Ser^253^ FoxO3a to total FoxO3a. PCSK9i treatment significantly enhanced AMPK phosphorylation, restored PGC-1α expression, and modulated FoxO1/3a signaling in *db*/*db* mice. Data are expressed as mean ± SD (*n* = 3 per group). Tukey-adjusted *p* for the indicated comparisons: * *p* < 0.05, ** *p* < 0.01, and *** *p* < 0.001.

To examine the involvement of AMPK-related mechanisms in the renoprotective effects of PCSK9 inhibition, AMPK phosphorylation and downstream signaling pathways were analyzed in *db*/*db* mice. The phospho-Thr^172^ AMPK/total AMPK ratio was significantly reduced, indicating suppressed AMPK activation in *db*/*db* mice (*p* = 0.002), and PCSK9 inhibition increased the phospho-Thr^172^ AMPK/total AMPK ratio in *db*/*db* mice by a mean difference of 1.29 (95% CI, 0.896–1.68; *p* < 0.001; Figure 3A,B). Consistently, PGC-1α expression was decreased in *db*/*db* mice and was significantly recovered following PCSK9i treatment (*p* = 0.04 and *p* < 0.001, respectively; Figure 3A,C). Diabetic mice also exhibited an increased phospho-Ser^473^ Akt/total Akt ratio, while PCSK9i treatment significantly attenuated Akt phosphorylation (both *p* < 0.001; Figure 3D,E). The ratios of phospho-Ser^256^ FoxO1/total FoxO1 and phospho-Ser^253^ FoxO3a/total FoxO3a were significantly increased in *db*/*db* mice (*p* = 0.03 and *p* = 0.006, respectively), and PCSK9i treatment effectively reduced these alterations (*p* = 0.007 and *p* = 0.004, respectively; Figure 3D,F,G).

### 3.4. PCSK9 Inhibition Modulates Lipogenesis and Fatty Acid Oxidation in Diabetic Mice

**Figure 4:**
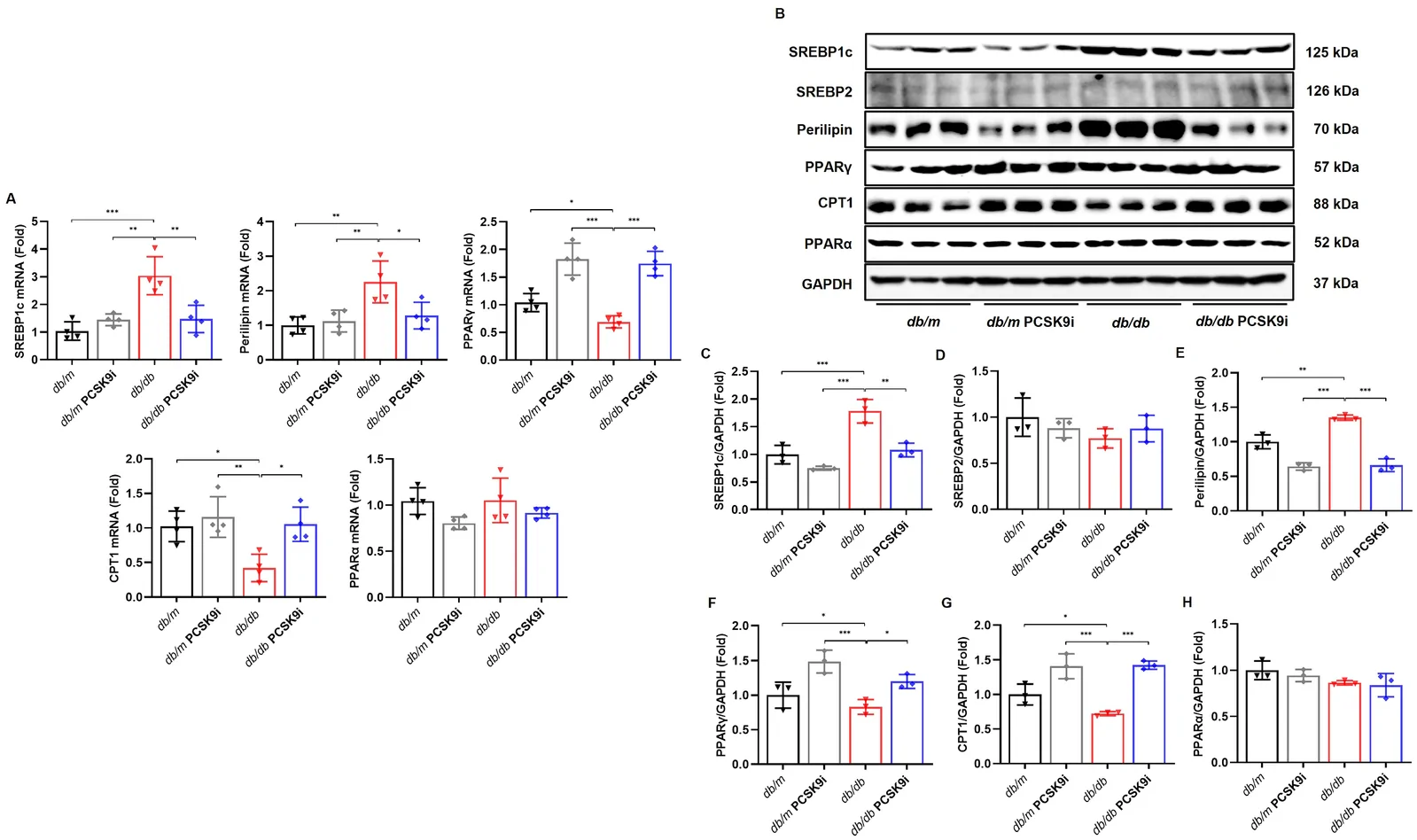
PCSK9 inhibition affects renal lipogenesis and fatty acid oxidation-related gene and protein levels. (**A**) Quantitative real-time PCR analysis of mRNA expression levels of SREBP-1c, perilipin, PPARα, CPT1, and PPARγ in kidney tissues. (**B**) Representative immunoblots showing protein expression of SREBP-1c, SREBP2, perilipin, PPARγ, CPT1, and PPARα in kidney tissues from *db*/*m* and *db*/*db* mice treated with or without PCSK9i. (**C**–**H**) Quantitative analyses of SREBP-1c, SREBP2, perilipin, PPARγ, CPT1, and PPARα. PCSK9i treatment significantly downregulated SREBP-1c and perilipin and restored CPT1 and PPARγ in *db*/*db* mice. Data are expressed as mean ± SD (mRNAs: *n* = 4 per group, immunoblots: *n* = 3 per group). Tukey-adjusted *p* for the indicated comparisons: * *p* < 0.05, ** *p* < 0.01, and *** *p* < 0.001.

Next, we examined the effects of PCSK9 inhibition on lipid metabolism in type 2 diabetic mice. In *db*/*db* mice, SREBP-1c and Perilipin mRNA levels were elevated (*p* < 0.001 and *p* = 0.007, respectively), while PPARγ and CPT1 mRNA levels were reduced (*p* = 0.048 and *p* = 0.02, respectively), with no significant change in PPARα. PCSK9i treatment reversed these alterations by downregulating SREBP-1c and Perilipin mRNA expression and restoring PPARγ and CPT1 mRNA levels (*p* = 0.002, *p* = 0.022, *p* < 0.001 and *p* = 0.015, respectively; Figure 4A). Consistent with the mRNA findings, SREBP-1c and Perilipin protein levels were markedly increased in *db*/*db* mice (*p* < 0.001 and *p* = 0.002, respectively) but decreased following PCSK9i treatment (*p* = 0.002 and *p* < 0.001, respectively), while SREBP2 protein expression remained unchanged (Figure 4B–E). Conversely, PPARγ and CPT1 protein levels were decreased in *db*/*db* mice (*p* = 0.048 and *p* = 0.02, respectively), but restored with PCSK9i treatment (*p* = 0.021 and *p* < 0.001, respectively), whereas PPARα protein expression remained unaffected (Figure 4B,F–H).

### 3.5. PCSK9 Inhibition Reduces Oxidative Stress and Apoptosis in Diabetic Mice

**Figure 5:**
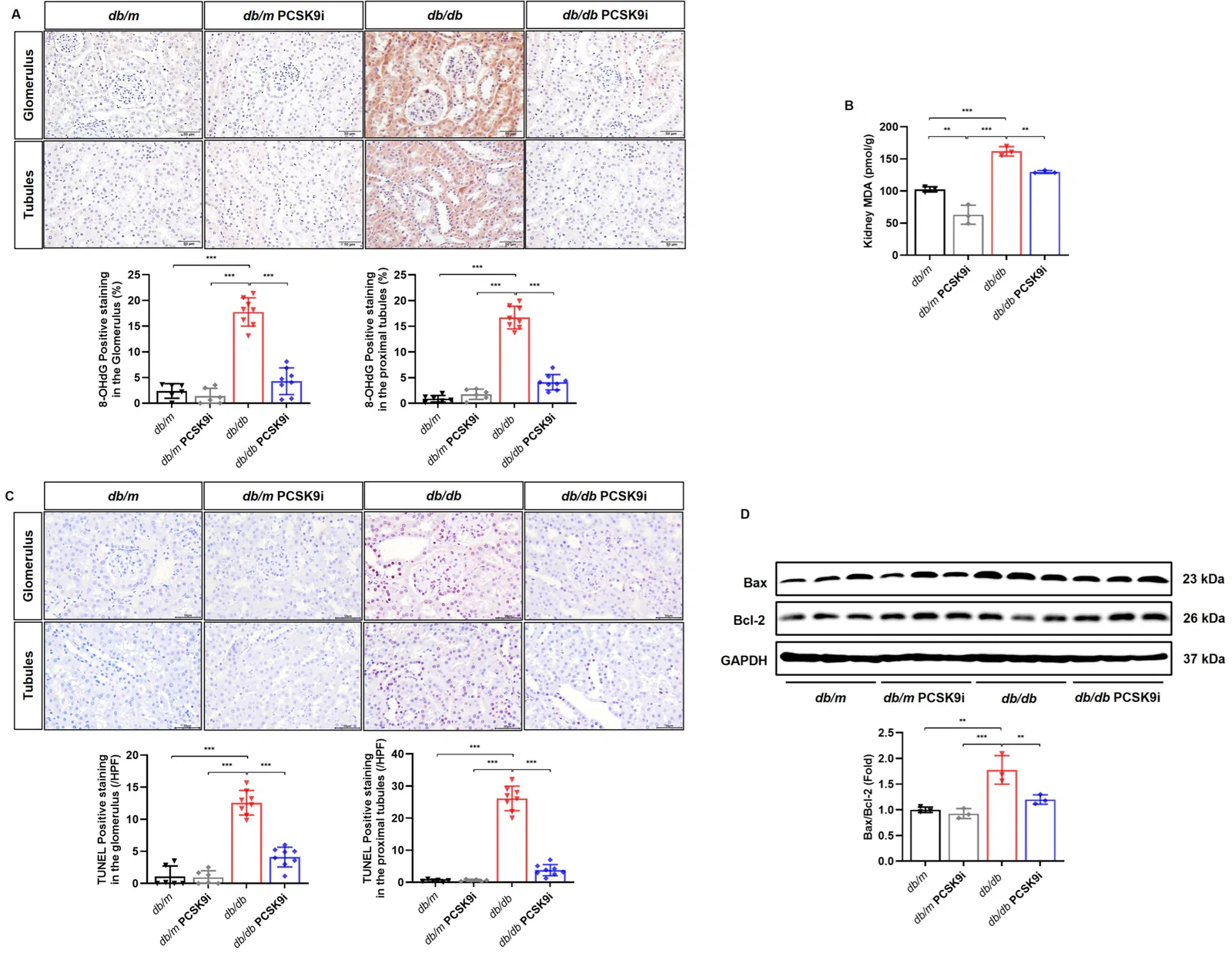
PCSK9 inhibition affects oxidative stress and apoptosis in the kidneys of *db*/*m* and *db*/*db* mice. (**A**) Representative immunohistochemical staining of 8-hydroxy-2′-deoxyguanosine (8-OHdG) in kidney tissues (original magnification ×400). Quantification of glomerular 8-OHdG-positive nuclei and tubular 8-OHdG staining demonstrated significantly increased oxidative stress in *db*/*db* mice, which was attenuated by PCSK9i treatment. (**B**) Intrarenal levels of malondialdehyde (MDA) were significantly elevated in *db*/*db* mice and reduced by PCSK9i. (**C**) Representative TUNEL staining and quantification of TUNEL-positive cells in the glomerulus and proximal tubules (original magnification ×400). PCSK9i treatment significantly reduced glomerular and tubular apoptosis in *db*/*db* mice. Data are expressed as mean ± SD (*n* = 6 per group: *db*/*m* and *db*/*m* + PCSK9i, *n* = 8 per group *db*/*db* and *db*/*db* + PCSK9i). (**D**) Representative immunoblots of Bax and Bcl-2 protein expression in kidney tissues. Quantitative analysis of the Bax/Bcl-2 protein expression ratio. The Bax/Bcl-2 ratio was significantly increased in *db*/*db* mice and attenuated by PCSK9i treatment. Data are expressed as mean ± SD (*n* = 3 per group). Tukey-adjusted *p* for the indicated comparisons: ** *p* < 0.01 and *** *p* < 0.001.

8-OHdG staining in the glomerulus and proximal tubules and kidney MDA levels were markedly elevated in *db*/*db* mice (all *p* < 0.001) and significantly reduced following PCSK9i treatment (*p* < 0.001, *p* < 0.001, and *p* = 0.008, respectively; Figure 5A,B), with a mean reduction in tubular 8-OHdG staining of 12.6% (95% CI, 10.5–14.7). The number of TUNEL-positive cells was significantly increased in *db*/*db* mice and attenuated by PCSK9i treatment in both the glomerulus and proximal tubules (both *p* < 0.001; Figure 5C), with a mean reduction of 22.3 tubular TUNEL-positive cells per field (95% CI, 19.2–25.5). In *db*/*db* mice, the anti-apoptotic protein Bcl-2 decreased, while the pro-apoptotic protein Bax increased, resulting in an elevated Bax/Bcl-2 ratio (*p* = 0.001). PCSK9i treatment enhanced Bcl-2 expression and reduced Bax levels in *db*/*db* mice, restoring the Bax/Bcl-2 ratio (*p* = 0.008; Figure 5D).

### 3.6. PCSK9 Inhibition Regulates Lipid Metabolism Through AMPK Signaling in HG-Treated HK-2 Cells

**Figure 6:**
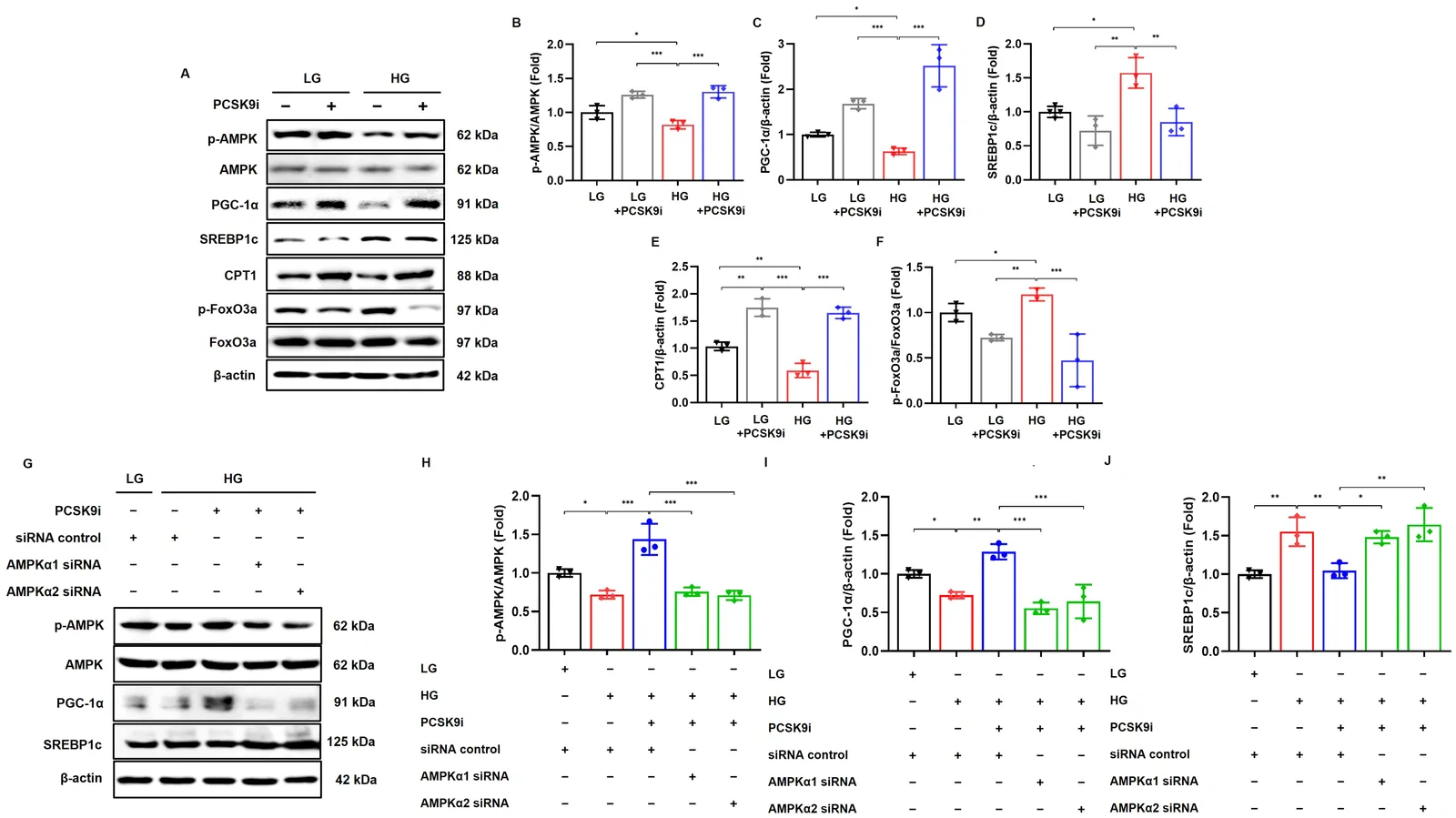
PCSK9 inhibition regulates AMPK-dependent lipid metabolism and antioxidant signaling in HG-treated HK-2 cells. (**A**) Representative immunoblots showing the expression of phosphorylated and total AMPK, PGC-1α, SREBP-1c, CPT1, and phosphorylated and total FoxO3a in HK-2 cells exposed to low glucose (LG) or high glucose (HG) with or without PCSK9i. (**B**–**F**) Quantitative analyses of phospho-Thr^172^ AMPK to total AMPK, PGC-1α, SREBP-1c, CPT1, and phospho-Ser^253^ FoxO3a to total FoxO3a. High glucose significantly suppressed AMPK phosphorylation, PGC-1α, and CPT1 expression, and increased SREBP-1c. These changes were reversed by PCSK9i treatment. (**G**) Representative immunoblots showing protein expression of phosphorylated and total AMPK, PGC-1α, and SREBP-1c in HK-2 cells cultured under LG or HG conditions, with or without PCSK9i. To investigate whether the effects of PCSK9 inhibition are mediated via AMPK signaling, cells were transfected with control siRNA or siRNA targeting either AMPKα1 or AMPKα2. (**H**–**J**) Quantitative analyses of phospho-Thr^172^ AMPK relative to total AMPK, PGC-1α and SREBP-1c. PCSK9i significantly increased AMPK phosphorylation and PGC-1α expression while decreasing SREBP-1c under HG conditions. These effects were abolished by either AMPKα1 or AMPKα2 knockdown, indicating AMPK-dependent mechanisms. Data are expressed as mean ± SD from three independent experiments (*n* = 3). Tukey-adjusted *p* for the indicated comparisons: * *p* < 0.05, ** *p* < 0.01, and *** *p* < 0.001.

To determine whether PCSK9 inhibition modulates AMPK-related signaling under hyperglycemic conditions, HK-2 cells were exposed to HG with or without PCSK9i treatment. HG induced marked decreases in the expression of phospho-Thr^172^ AMPK/total AMPK and PGC-1α (*p* = 0.047 and *p* = 0.049, respectively), and PCSK9 inhibition restored AMPK phosphorylation and PGC-1α in HG-treated HK-2 cells (all *p* < 0.001; Figure 6A–C). The expression of SREBP-1c and phospho-Ser^253^/total FoxO3a significantly increased (*p* = 0.032 and *p* = 0.047, respectively), while the expression of CPT1 was decreased in HG-treated HK-2 cells (*p* = 0.02). These alterations were significantly improved following treatment with PCSK9i (*p* = 0.006, *p* < 0.001, and *p* < 0.001, respectively; Figure 6A,D–F). Consistent with the effects of SBC-115076, both evolocumab treatment and PCSK9 silencing increased AMPK phosphorylation and PGC-1α expression while decreasing SREBP-1c expression in HG-treated HK-2 cells (Supplementary Figure S4).

To further investigate whether these effects are mediated via AMPK signaling, HK-2 cells were transfected with siRNAs targeting *AMPKα1* and *AMPKα2*. As shown in Figure 6G,H, the phospho-Thr^172^ AMPK/total AMPK ratios were significantly reduced in HG-treated HK-2 cells following either AMPKα1 or AMPKα2 knockdown, despite PCSK9i treatment (all *p* < 0.001). Consistent with the reduction in AMPK expression, the expression of PGC-1α also markedly decreased under the same conditions (*p* < 0.001; Figure 6G,I). In contrast, the expression of SREBP-1c was significantly increased in HG-treated HK-2 cells with either AMPKα1 or AMPKα2 knockdown compared to the siRNA control group, even with PCSK9i treatment (*p* = 0.024 and *p* = 0.003, respectively; Figure 6G,J).

### 3.7. PCSK9 Inhibition Reduces Lipid Accumulation and Oxidative Stress Through AMPK Signaling in HG-Treated HK-2 Cells

**Figure 7:**
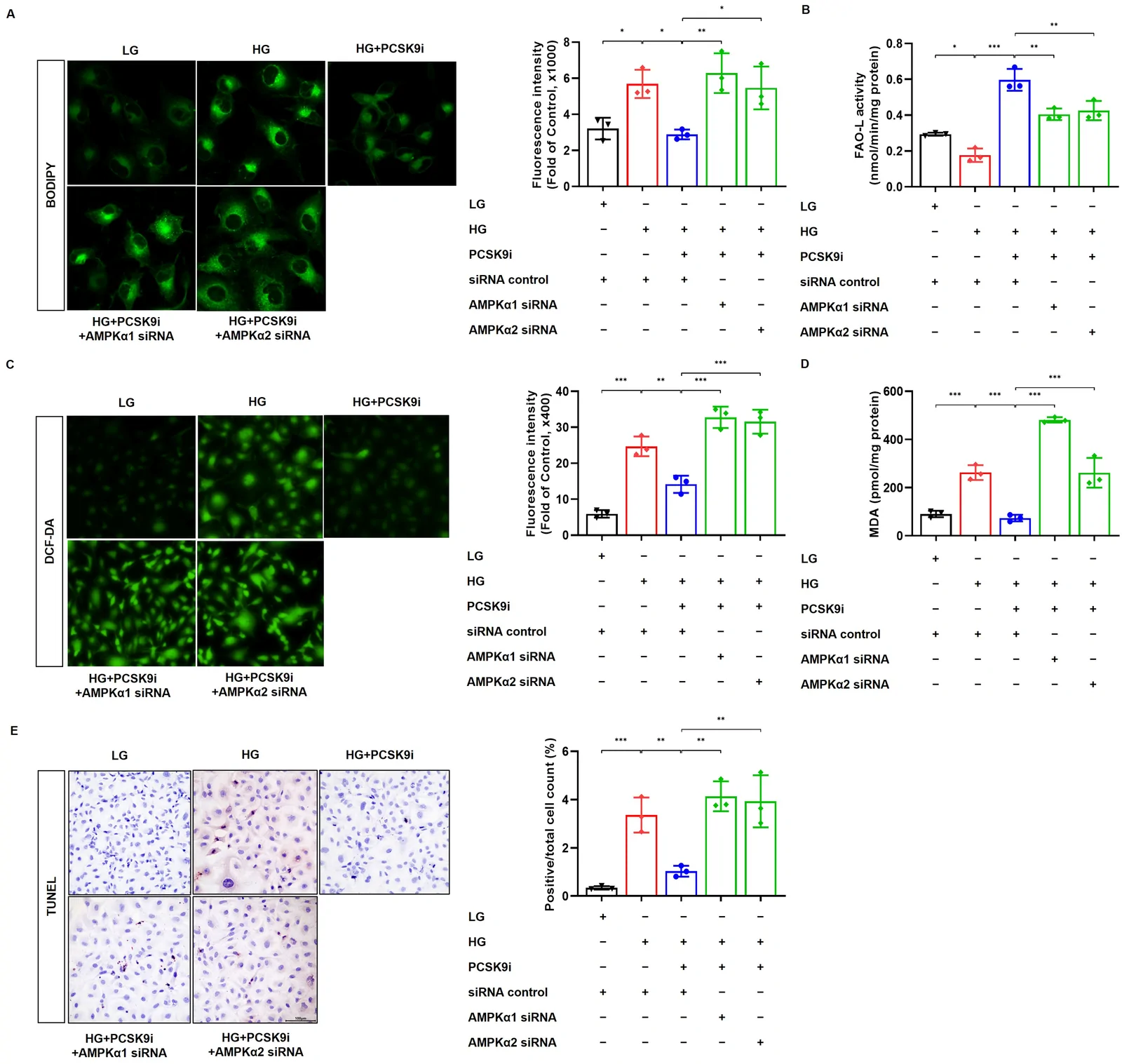
AMPK knockdown reverses the protective effects of PCSK9 inhibition against HG-induced oxidative stress and apoptosis. (**A**) Representative BODIPY staining and quantification of intracellular lipid accumulation in HK-2 cells cultured under LG or HG conditions, with or without PCSK9i, and transfected with control siRNA or siRNAs targeting AMPKα1 or AMPKα2. (**B**) Long-chain fatty acid oxidation (FAO-L) activity was significantly reduced in HG-exposed cells. PCSK9i reversed this reduction, whereas either AMPKα1 or AMPKα2 knockdown significantly attenuated the PCSK9i-induced increase in FAO-L activity. (**C**) Intracellular oxidative stress assessed using DCF-DA staining. PCSK9i markedly reduced HG-induced oxidative stress assessed by DCF-DA staining, which was reversed by either AMPKα1 or AMPKα2 knockdown. (**D**) Malondialdehyde (MDA) levels, an index of lipid peroxidation, were decreased by PCSK9i treatment but restored by AMPK knockdown. (**E**) TUNEL staining revealed that PCSK9i significantly reduced HG-induced apoptosis, and this effect was abolished by either AMPKα1 or AMPKα2 knockdown. Data are expressed as mean ± SD from three independent experiments (*n* = 3). Tukey-adjusted *p* for the indicated comparisons: * *p* < 0.05, ** *p* < 0.01, and *** *p* < 0.001.

BODIPY staining demonstrated that PCSK9i reduced HG-induced lipid accumulation in HK-2 cells (*p* = 0.02); however, this effect was abolished by either AMPKα1 or AMPKα2 knockdown (*p* = 0.004 and *p* = 0.03, respectively; Figure 7A). Long-chain FAO activity was significantly reduced under HG conditions (*p* = 0.045), increased by PCSK9 inhibition (*p* < 0.001), and this increase was significantly attenuated by either AMPKα1 or AMPKα2 knockdown (*p* = 0.002 and *p* = 0.004, respectively; Figure 7B). HG increased intracellular oxidative stress and lipid peroxidation, as indicated by DCF-derived fluorescence intensity and MDA levels, respectively (both *p* < 0.001). These redox-related parameters were significantly reduced by PCSK9i treatment (*p* = 0.004 and *p* < 0.001, respectively). However, the antioxidant effects of PCSK9i were abolished in HG-treated cells transfected with either *PRKAA1* or *PRKAA2* siRNAs (all *p* < 0.001; Figure 7C,D). Similarly, HG increased the number of TUNEL-positive apoptotic cells (*p* < 0.001), while PCSK9i treatment reduced apoptosis in HK-2 cells (*p* = 0.009). This antiapoptotic effect was abolished by either AMPKα1 or AMPKα2 knockdown in HG-treated HK-2 cells (*p* = 0.001 and *p =* 0.002; Figure 7E).

## 4. Discussion

The present study demonstrates that PCSK9 inhibition attenuates DKD by alleviating renal lipotoxicity in association with AMPK activation. PCSK9 inhibition enhanced AMPK phosphorylation and was accompanied by changes in downstream signaling, suppressed lipogenesis, improved lipid homeostasis, and reduced oxidative stress and apoptosis in both in vivo and in vitro DKD models. These findings extend the role of PCSK9 beyond systemic lipid regulation and identify it as a modulator of renal metabolic stress in DKD. In addition, our data suggest that PCSK9 inhibition is associated with the regulation of renal redox homeostasis and the interplay between lipid metabolism and oxidative stress pathways.

Recent experimental studies have demonstrated the renoprotective potential of PCSK9 inhibition in various kidney injury models. In cisplatin-induced acute kidney injury, evolocumab reduced proteinuria and preserved megalin expression by preventing its lysosomal degradation [18]. Anti-PCSK9 vaccination ameliorated renal lipid accumulation and fibrosis in experimental renal fibrosis models [19]. PCSK9 inhibition also attenuated podocytopathy by suppressing inflammatory signaling, including the NOD-like receptor protein 3 (NLRP3) inflammasome pathway and the cyclic GMP-AMP synthase (cGAS)/stimulator of interferon genes (STING) pathway in models of nephrotic syndrome and DKD [20,21]. Similarly, alirocumab reduced proteinuria by upregulating megalin expression in podocin-knockout mice [14]. Previous studies have primarily focused on inflammatory and fibrotic mechanisms, whereas our findings highlight an association between AMPK-related signaling and renal lipid metabolism following PCSK9 inhibition in type 2 DKD.

A notable finding of this study is that genetic deficiency and pharmacological inhibition of PCSK9 may exert distinct effects on renal lipid handling. In adriamycin nephropathy and diabetic models, renal PCSK9 expression was reduced, whereas renal lipid accumulation was increased [22,23]. Similarly, PCSK9 knockout in a DKD model was associated with upregulation of LDLR and CD36, accompanied by increased glomerular lipid accumulation and podocyte injury [23]. Moreover, PCSK9 knockout mice fed a high-fat diet exhibited tubular lipid accumulation, whereas pharmacological inhibition with evolocumab reduced CD36 expression, thereby attenuating ER stress, inflammation, and fibrosis [24]. This discrepancy suggests that the functional activity of PCSK9 may not be solely determined by its local expression levels, but rather by its systemic availability and interaction with lipid transport pathways. In our study, although renal PCSK9 expression was suppressed in *db*/*db* mice, pharmacological PCSK9 inhibition reduced CD36 expression, restored lipid homeostasis, and ameliorated diabetic kidney injury, underscoring the functional differences between genetic ablation and pharmacological inhibition.

To date, the relationship between PCSK9 inhibition and AMPK signaling in renal metabolic regulation remains incompletely understood. Previous studies have linked PCSK9 inhibition to AMPK activation primarily through autophagy-related pathways [25,26]. For example, PCSK9 inhibition reduced cardiac ischemic damage by limiting autophagy via the ROS–liver kinase B1 (LKB1)–AMPK axis [25]. Anti-PCSK9 treatment also ameliorated hepatic inflammation and fibrosis by suppressing hypoxia-induced autophagy through the AMPK/mechanistic target of rapamycin (mTOR)/UNC-51-like kinase 1 (ULK1) pathway [26]. In our study, PCSK9 inhibition attenuated lipid accumulation and oxidative stress and was accompanied by enhanced AMPK signaling, and AMPK knockdown in HK-2 cells further supported a functional role for AMPK in these effects. Importantly, whereas previous studies emphasized the role of AMPK in autophagy- and fibrosis-related pathways, our findings identify lipid metabolism and redox homeostasis as downstream processes functionally linked to AMPK signaling following PCSK9 inhibition.

Proximal tubular cells are mitochondria-rich and highly dependent on ATP generation to sustain active solute reabsorption [27]. The pathophysiological paradigm of DKD has shifted from a glomerulocentric view to the concept of diabetic tubulopathy [28,29]. Lipotoxicity disrupts mitochondrial function and impairs FAO, resulting in fatty acid accumulation, excess ROS production, and tubular cell apoptosis [30]. These changes are characterized by increased CD36-mediated fatty acid uptake, reduced β-oxidation due to downregulation of PPARs, PGC-1α, and CPT1, and enhanced lipogenesis driven by SREBP [31]. We found that PCSK9 expression was predominantly localized to proximal tubules and was accompanied by increased SREBP-1c and decreased PGC-1α and CPT1. PCSK9 inhibition reversed these alterations in parallel with restoration of AMPK signaling. Consistently, tubular injury markers, including NGAL and KIM-1 [32], were elevated in *db*/*db* mice and significantly reduced by PCSK9 inhibition. In HG-treated HK-2 cells, both CPT1 expression and long-chain FAO activity were reduced, and PCSK9 inhibition reversed these reductions, whereas either AMPKα1 or AMPKα2 knockdown attenuated the increase in long-chain FAO activity induced by PCSK9 inhibition. These findings support the involvement of AMPK signaling in the restoration of FAO and lipid homeostasis by PCSK9 inhibition. PCSK9 inhibition attenuated this lipotoxicity–ROS axis and was associated with improved metabolic and redox homeostasis in proximal tubular cells.

Emerging evidence indicates that dynamic cellular communication among various kidney cell types critically orchestrates the progression of DKD [33,34]. In particular, tubule-derived metabolic and inflammatory signals have been shown to modulate glomerular structure and function through intercellular crosstalk mechanisms [34]. In this study, PCSK9 inhibition attenuated albuminuria, mesangial expansion, and the expression of TGF-β1 and type IV collagen. Our findings suggest that AMPK-related metabolic effects of PCSK9 inhibition in proximal tubular cells may influence glomerular remodeling, further supporting a potential role for proximal tubular metabolism in tubuloglomerular crosstalk during DKD progression.

The therapeutic potential of AMPK activation in DKD has been well established [35,36]. AMPK activation improved glucose and lipid metabolism, reduced oxidative stress, and prevented renal hypertrophy in DKD [36,37]. Specifically, AMPK attenuated lipotoxicity by suppressing SREBP-1c-driven lipogenesis and promoting CPT1-mediated fatty acid oxidation [37]. Several pharmacological and natural compounds have been shown to ameliorate lipotoxicity in DKD. For example, resveratrol and anthocyanins activated AMPK–SIRT1–PGC-1α signaling and reduced lipotoxicity [38,39]. Fenofibrate stimulated AMPK–PGC-1α–estrogen-related receptor α (ERRα)–FoxO3a signaling and improved diabetic kidney injury [7]. AdipoRon enhanced AMPK–Akt–endothelial nitric oxide synthase (eNOS) and PPARα–Akt–eNOS pathways, while also reducing ceramide accumulation [9]. The present study suggests that PCSK9 inhibition may represent an additional therapeutic strategy for activating AMPK signaling in DKD.

PGC-1α is a pivotal transcriptional coactivator that regulates mitochondrial biogenesis, oxidative phosphorylation, carbohydrate and lipid energy metabolism, and cellular defense against ROS [40]. AMPK directly activates PGC-1α and modulates FoxO signaling either directly or indirectly through upstream regulators such as Akt, thereby enhancing mitochondrial function and resistance to oxidative stress [41,42,43]. In addition, PGC-1α interacts with FoxO1 and FoxO3a to coordinate antioxidant defense [44]. In our study, PCSK9 inhibition increased PGC-1α expression and reduced FoxO phosphorylation, while AMPK knockdown abolished the PCSK9i-induced increase in PGC-1α expression and the antioxidant effects of PCSK9 inhibition in HG-treated HK-2 cells. These findings suggest that modulation of AMPK–PGC-1α and FoxO signaling is associated with the reduced oxidative stress observed following PCSK9 inhibition in DKD.

This study has several limitations. First, the bioavailability, stability, renal distribution, and target engagement of SBC-115076 in the kidney were not directly evaluated, and circulating PCSK9 concentrations were not measured. Moreover, because SBC-115076 improved systemic lipid profiles and hepatic steatosis, its direct renal effects cannot be distinguished from those secondary to systemic metabolic improvement, and potential off-target effects cannot be excluded. Second, AMPK knockdown supported AMPK as a functional downstream mediator of PCSK9 inhibition but did not establish a direct molecular interaction between PCSK9 and AMPK or its causal role in vivo. Furthermore, potential siRNA off-target effects cannot be excluded because independent siRNAs or rescue experiments were not performed. Third, the cellular experiments were limited to HK-2 cells and did not examine other renal cell types involved in DKD. Fourth, the lysate-based FAO assay reflects enzymatic activity rather than real-time fatty-acid oxidation or mitochondrial respiratory flux in intact cells. Furthermore, general redox markers such as DCF-DA, MDA, and 8-OHdG were used without direct evaluation of mitochondrial ROS production, antioxidant enzyme activities, NRF2 signaling, or mitochondrial respiration, and FoxO nuclear localization, transcriptional activity and downstream targets were not assessed. Finally, SBC-115076 may differ from clinically available anti-PCSK9 monoclonal antibodies in its pharmacological properties and tissue accessibility, warranting further validation of these findings using clinically available PCSK9-directed therapies.

## 5. Conclusions

**Figure 8:**
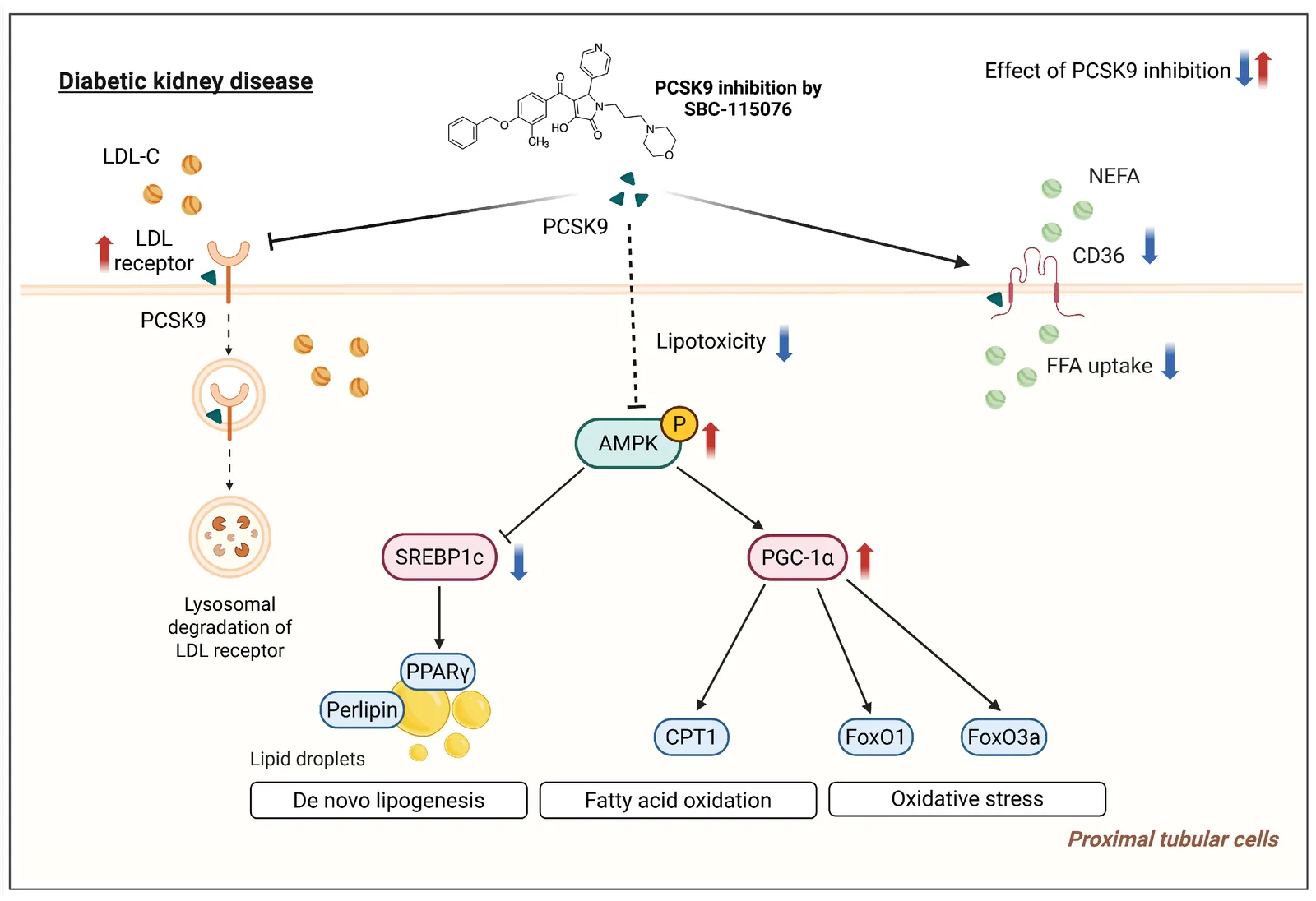
Proposed mechanisms by which PCSK9 inhibition may attenuate renal lipotoxicity in diabetic kidney disease. PCSK9 inhibition was associated with increased LDLR expression and decreased CD36 expression, consistent with reduced lipid accumulation in renal proximal tubular cells. It was also associated with increased AMPK phosphorylation and PGC-1α expression, accompanied by reduced FoxO1/3a phosphorylation and oxidative stress. In addition, PCSK9 inhibition was associated with downregulation of SREBP-1c and perilipin and increased CPT1 and PPARγ expression. Collectively, these molecular changes were associated with reduced lipid accumulation, oxidative stress, and apoptosis, as well as attenuated glomerular injury and albuminuria, in diabetic kidney disease. Red upward and blue downward arrows indicate increases and decreases, respectively, following PCSK9 inhibition. (Created in BioRender. Hong, Y. A. (2026) https://BioRender.com/y8drdkd).

Our study demonstrated that PCSK9 inhibition attenuated renal lipotoxicity, oxidative stress, and apoptosis in experimental models of DKD in association with AMPK activation (Figure 8). PCSK9 inhibition was also associated with increased PGC-1α expression, suppression of SREBP-1c signaling, increased CPT1 expression, and reduced FoxO phosphorylation. Furthermore, *AMPKα1*/*α2* knockdown attenuated the protective effects of PCSK9 inhibition in HK-2 cells, supporting the functional involvement of AMPK signaling. Given the clinical availability of PCSK9-directed therapies [45,46], our findings provide a preclinical rationale for further investigating whether these therapies exert renal metabolic effects beyond their established systemic lipid-lowering effects.

## Figures and Tables

**Table 1:** Physical and biochemical parameters of the experimental groups.

	*db*/*m* Control	*db*/*m* PCSK9i	*db*/*db* Control	*db*/*db* PCSK9i
BW (g)	26.3 ± 2.6	25.9 ± 1.9	46.4 ± 8.0 ***	43.4 ± 7.9 ***
KW/BW (g/100 g)	1.34 ± 0.10	1.44 ± 0.14	1.22 ± 0.39	1.08 ± 0.1
FBS (mg/dL)	128.7 ± 30.3	121.2 ± 16.1	686.7 ± 218.8 ***	682.0 ± 224.0 ***
HbA1c (%)	3.9 ± 0.3	4.1 ± 0.3	10.1 ± 1.4 ***	10.6 ± 1.1 ***
HOMA-IR	1.99 ± 0.76	1.56 ± 0.68	12.44 ± 6.32 ***	16.59 ± 5.52 ***
AST (IU/L)	199.1 ± 61.6	214.4 ± 42.5	240.1 ± 46.7 *	174.0 ± 65.6 ^†††^
ALT (IU/L)	91.4 ± 35.2	107.2 ± 31.3	219.6 ± 56.0 ***	119.0 ± 46.6 ^†††^
Serum cystatin C (ng/mL)	186.8 ± 19.2	186.4 ± 17.9	189.9 ± 5.6	192.1 ± 11.8
Serum KIM-1 (pg/mL)	47.9 ± 8.1	43.8 ± 6.1	155.3 ± 50.0 ***	53.8 ± 21.0 ^†††^
Serum NGAL (ng/mL)	25.0 ± 7.9	35.4 ± 8.9	532.4 ± 198.4 ***	144.1 ± 30.9 ^†††^

KW, kidney weight; BW, body weight; FBS, fasting blood glucose; HbA1c, hemoglobin A1c; HOMA-IR, homeostatic model assessment for insulin resistance; AST, aspartate aminotransferase; ALT, alanine aminotransferase; KIM-1, kidney injury molecule-1; NGAL, neutrophil gelatinase-associated lipocalin. Data are expressed as mean ± SD (*n* = 6 per group: *db*/*m* and *db*/*m* + PCSK9i, *n* = 8 per group *db*/*db* and *db*/*db* + PCSK9i). Tukey-adjusted *p* values: * *p* < 0.05 and *** *p* < 0.001 vs. the *db/m* control group; ^†††^ *p* < 0.001 vs. the *db/db* control group.

## Data Availability

The original contributions presented in this study are included in the article/Supplementary Materials. Further inquiries can be directed to the corresponding author.

## Supplementary Materials

The following supporting information can be downloaded at: https://www.mdpi.com/article/10.3390/antiox15091182/s1. Supplementary Methods: Effects of evolocumab treatment and PCSK9 silencing in HG-treated HK-2 cells; Figure S1: Effect of SBC-115076 on HK-2 cell viability; Figure S2: Time-course validation of the efficiency and isoform selectivity of AMPKα1 and AMPKα2 knockdown in HK-2 cells; Figure S3: Changes in histological findings of the liver in db/db mice treated with PCSK9 inhibitors; Figure S4: Effects of evolocumab and PCSK9 silencing on AMPK-related signaling in high-glucose-treated HK-2 cells; Table S1: Commercial assays and ELISA kits; Table S2: Antibodies used in Western blotting and immunohistochemistry; Table S3: siRNA sequences; Table S4: Primer sequences for qRT-PCR.

## Abbreviations

AMPK	AMP-activated protein kinase
Bax	Bcl-2 associated X
Bcl-2	B-cell lymphoma 2
CD36	cluster of differentiation 36
CPT1	carnitine palmitoyltransferase 1
DKD	diabetic kidney disease
FAO	fatty acid oxidation
FoxO1	forkhead box O1
FoxO3a	forkhead box O3a
GAPDH	glyceraldehyde 3-phosphate dehydrogenase
HG	high glucose
HK-2	human kidney-2
KIM-1	kidney injury molecule-1
LDLR	low-density lipoprotein receptor
LG	low glucose
MDA	malondialdehyde
NEFA	non-esterified fatty acid
NGAL	neutrophil gelatinase-associated lipocalin
PCSK9	proprotein convertase subtilisin/kexin type 9
PCSK9i	PCSK9 inhibitor
PGC-1α	peroxisome proliferator-activated receptor gamma coactivator 1-alpha
PPARα	peroxisome proliferator-activated receptor alpha
PPARγ	peroxisome proliferator-activated receptor gamma
ROS	reactive oxygen species
SREBP	sterol regulatory element-binding protein
TGF-β1	transforming growth factor-β1
TUNEL	terminal deoxynucleotidyl transferase-mediated dUTP nick-end labeling

## References

[1] Hong Y.A., Ban T.H., Kang C.Y., Hwang S.D., Choi S.R., Lee H., Jung H.Y., Kim K., Kwon Y.E., Kim S.H. (2021). Trends in epidemiologic characteristics of end-stage renal disease from 2019 Korean Renal Data System (KORDS). Kidney Res. Clin. Pract..

[2] DeFronzo R.A., Reeves W.B., Awad A.S. (2021). Pathophysiology of diabetic kidney disease: Impact of SGLT2 inhibitors. Nat. Rev. Nephrol..

[3] de Vries A.P., Ruggenenti P., Ruan X.Z., Praga M., Cruzado J.M., Bajema I.M., D’Agati V.D., Lamb H.J., Pongrac Barlovic D., Hojs R. (2014). Fatty kidney: Emerging role of ectopic lipid in obesity-related renal disease. Lancet Diabetes Endocrinol..

[4] Garcia D., Shaw R.J. (2017). AMPK: Mechanisms of cellular energy sensing and restoration of metabolic balance. Mol. Cell.

[5] Steinberg G.R., Hardie D.G. (2023). New insights into activation and function of the AMPK. Nat. Rev. Mol. Cell Biol..

[6] Hardie D.G., Schaffer B.E., Brunet A. (2016). AMPK: An Energy-Sensing Pathway with Multiple Inputs and Outputs. Trends Cell Biol..

[7] Hong Y.A., Lim J.H., Kim M.Y., Kim T.W., Kim Y., Yang K.S., Park H.S., Choi S.R., Chung S., Kim H.W. (2014). Fenofibrate improves renal lipotoxicity through activation of AMPK-PGC-1α in *db*/*db* mice. PLoS ONE.

[8] Kim Y., Lim J.H., Kim M.Y., Kim E.N., Yoon H.E., Shin S.J., Choi B.S., Kim Y.S., Chang Y.S., Park C.W. (2018). The Adiponectin Receptor Agonist AdipoRon Ameliorates Diabetic Nephropathy in a Model of Type 2 Diabetes. J. Am. Soc. Nephrol..

[9] Choi S.R., Lim J.H., Kim M.Y., Kim E.N., Kim Y., Choi B.S., Kim Y.S., Kim H.W., Lim K.M., Kim M.J. (2018). Adiponectin receptor agonist AdipoRon decreased ceramide, and lipotoxicity, and ameliorated diabetic nephropathy. Metabolism.

[10] Seidah N.G., Benjannet S., Wickham L., Marcinkiewicz J., Jasmin S.B., Stifani S., Basak A., Prat A., Chretien M. (2003). The secretory proprotein convertase neural apoptosis-regulated convertase 1 (NARC-1): Liver regeneration and neuronal differentiation. Proc. Natl. Acad. Sci. USA.

[11] Zaid A., Roubtsova A., Essalmani R., Marcinkiewicz J., Chamberland A., Hamelin J., Tremblay M., Jacques H., Jin W., Davignon J. (2008). Proprotein convertase subtilisin/kexin type 9 (PCSK9): Hepatocyte-specific low-density lipoprotein receptor degradation and critical role in mouse liver regeneration. Hepatology.

[12] Haas M.E., Levenson A.E., Sun X., Liao W.H., Rutkowski J.M., de Ferranti S.D., Schumacher V.A., Scherer P.E., Salant D.J., Biddinger S.B. (2016). The Role of Proprotein Convertase Subtilisin/Kexin Type 9 in Nephrotic Syndrome-Associated Hypercholesterolemia. Circulation.

[13] Feng Z., Liao X., Zhang H., Peng J., Huang Z., Yi B. (2023). Increased serum PCSK9 levels are associated with renal function impairment in patients with type 2 diabetes mellitus. Ren. Fail..

[14] Skeby C.K., Hummelgaard S., Gustafsen C., Petrillo F., Frederiksen K.P., Olsen D., Kristensen T., Ivarsen P., Madsen P., Christensen E.I. (2023). Proprotein convertase subtilisin/kexin type 9 targets megalin in the kidney proximal tubule and aggravates proteinuria in nephrotic syndrome. Kidney Int..

[15] Molina-Jijon E., Gambut S., Macé C., Avila-Casado C., Clement L.C. (2020). Secretion of the epithelial sodium channel chaperone PCSK9 from the cortical collecting duct links sodium retention with hypercholesterolemia in nephrotic syndrome. Kidney Int..

[16] Meyerholz D.K., Beck A.P. (2018). Principles and approaches for reproducible scoring of tissue stains in research. Lab. Investig..

[17] Bligh E.G., Dyer W.J. (1959). A rapid method of total lipid extraction and purification. Can. J. Biochem. Physiol..

[18] Wang Z., Yu X., Ma H., Yao S., Li Z., Zhang R., Liang H., Jiao J. (2025). Proprotein convertase subtilisin/kexin type 9 contributes to cisplatin-induced acute kidney injury by interacting with cyclase-associated protein 1 to promote megalin lysosomal degradation. Biochim. Biophys. Acta Mol. Cell Res..

[19] Wu D., Zhou Y., Pan Y., Li C., Wang Y., Chen F., Chen X., Yang S., Zhou Z., Liao Y. (2020). Vaccine Against PCSK9 Improved Renal Fibrosis by Regulating Fatty Acid β-Oxidation. J. Am. Heart Assoc..

[20] Suzuki T., Iyoda M., Kanazawa N., Tachibana S., Honda H. (2023). Effect of Proprotein Convertase Subtilisin/Kexin Type 9 Inhibition on Podocytes in Mouse Nephrotic Syndrome. Lab. Investig..

[21] Feng Z., Liao X., Peng J., Quan J., Zhang H., Huang Z., Yi B. (2023). PCSK9 causes inflammation and cGAS/STING pathway activation in diabetic nephropathy. FASEB J..

[22] Zhang G., Li Q. (2019). Inflammation Induces Lipid Deposition in Kidneys by Downregulating Renal PCSK9 in Mice with Adriamycin-Induced Nephropathy. Med. Sci. Monit..

[23] Wu M., Yoon C.Y., Park J., Kim G., Nam B.Y., Kim S., Park J.T., Han S.H., Kang S.W., Yoo T.H. (2024). The role of PCSK9 in glomerular lipid accumulation and renal injury in diabetic kidney disease. Diabetologia.

[24] Byun J.H., Lebeau P.F., Platko K., Carlisle R.E., Faiyaz M., Chen J., MacDonald M.E., Makda Y., Yousof T., Lynn E.G. (2022). Inhibitory Antibodies against PCSK9 Reduce Surface CD36 and Mitigate Diet-Induced Renal Lipotoxicity. Kidney360.

[25] Ding Z., Wang X., Liu S., Shahanawaz J., Theus S., Fan Y., Deng X., Zhou S., Mehta J.L. (2018). PCSK9 expression in the ischaemic heart and its relationship to infarct size, cardiac function, and development of autophagy. Cardiovasc. Res..

[26] Ning L., Zou Y., Li S., Cao Y., Xu B., Zhang S., Cai Y. (2023). Anti-PCSK9 Treatment Attenuates Liver Fibrosis via Inhibiting Hypoxia-Induced Autophagy in Hepatocytes. Inflammation.

[27] Bhargava P., Schnellmann R.G. (2017). Mitochondrial energetics in the kidney. Nat. Rev. Nephrol..

[28] Tang S.C., Lai K.N. (2012). The pathogenic role of the renal proximal tubular cell in diabetic nephropathy. Nephrol. Dial. Transplant..

[29] Gilbert R.E. (2017). Proximal Tubulopathy: Prime Mover and Key Therapeutic Target in Diabetic Kidney Disease. Diabetes.

[30] Sakashita M., Tanaka T., Inagi R. (2021). Metabolic Changes and Oxidative Stress in Diabetic Kidney Disease. Antioxidants.

[31] Wang H., Zhang S., Guo J. (2021). Lipotoxic Proximal Tubular Injury: A Primary Event in Diabetic Kidney Disease. Front. Med..

[32] Satirapoj B. (2018). Tubulointerstitial Biomarkers for Diabetic Nephropathy. J. Diabetes Res..

[33] Lv L., Zheng W., Hu M., Cheing G.L.Y., Liu Z., Zhou S., Cheung A.K.K. (2026). The Role of Cell-Cell Communication in Renal Damage and the Therapeutic Targeting of Diabetic Kidney Disease. Am. J. Nephrol..

[34] Jiang S., Su H. (2023). Cellular crosstalk of mesangial cells and tubular epithelial cells in diabetic kidney disease. Cell Commun. Signal..

[35] Ayinde K.S., Olaoba O.T., Ibrahim B., Lei D., Lu Q., Yin X., Adelusi T.I. (2020). AMPK allostery: A therapeutic target for the management/treatment of diabetic nephropathy. Life Sci..

[36] Kim Y., Park C.W. (2016). Adenosine monophosphate-activated protein kinase in diabetic nephropathy. Kidney Res. Clin. Pract..

[37] Juszczak F., Caron N., Mathew A.V., Declèves A.E. (2020). Critical Role for AMPK in Metabolic Disease-Induced Chronic Kidney Disease. Int. J. Mol. Sci..

[38] Koh E.S., Lim J.H., Kim M.Y., Chung S., Shin S.J., Choi B.S., Kim H.W., Hwang S.Y., Kim S.W., Park C.W. (2015). Anthocyanin-rich Seoritae extract ameliorates renal lipotoxicity via activation of AMP-activated protein kinase in diabetic mice. J. Transl. Med..

[39] Kim M.Y., Lim J.H., Youn H.H., Hong Y.A., Yang K.S., Park H.S., Chung S., Ko S.H., Shin S.J., Choi B.S. (2013). Resveratrol prevents renal lipotoxicity and inhibits mesangial cell glucotoxicity in a manner dependent on the AMPK-SIRT1-PGC1α axis in *db*/*db* mice. Diabetologia.

[40] Liang H., Ward W.F. (2006). PGC-1alpha: A key regulator of energy metabolism. Adv. Physiol. Educ..

[41] Cantó C., Auwerx J. (2009). PGC-1alpha, SIRT1 and AMPK, an energy sensing network that controls energy expenditure. Curr. Opin. Lipidol..

[42] Greer E.L., Banko M.R., Brunet A. (2009). AMP-activated protein kinase and FoxO transcription factors in dietary restriction-induced longevity. Ann. N. Y. Acad. Sci..

[43] Davila D., Connolly N.M., Bonner H., Weisová P., Dussmann H., Concannon C.G., Huber H.J., Prehn J.H. (2012). Two-step activation of FOXO3 by AMPK generates a coherent feed-forward loop determining excitotoxic cell fate. Cell Death Differ..

[44] Klotz L.O., Sánchez-Ramos C., Prieto-Arroyo I., Urbánek P., Steinbrenner H., Monsalve M. (2015). Redox regulation of FoxO transcription factors. Redox Biol..

[45] Robinson J.G., Farnier M., Krempf M., Bergeron J., Luc G., Averna M., Stroes E.S., Langslet G., Raal F.J., El Shahawy M. (2015). Efficacy and safety of alirocumab in reducing lipids and cardiovascular events. N. Engl. J. Med..

[46] Sabatine M.S., Giugliano R.P., Wiviott S.D., Raal F.J., Blom D.J., Robinson J., Ballantyne C.M., Somaratne R., Legg J., Wasserman S.M. (2015). Efficacy and safety of evolocumab in reducing lipids and cardiovascular events. N. Engl. J. Med..

