# Supplementary material for: PCSK9 Inhibition Attenuates Renal Oxidative Stress and Lipotoxicity in Diabetic Kidney Disease in Association with AMPK Activation
Source: Antioxidants (Basel). 2026 Sep 17;15(9):1182. doi: 10.3390/antiox15091182 (PMC13603972; doi:10.3390/antiox15091182)
Supplement: Supplementary file 1 [file antioxidants-15-01182-s001.zip › antioxidants-4462682-supplementary.pdf]

Supplementary Methods

Effects of evolocumab treatment and PCSK9 silencing in HG-treated HK-2 cells

HK-2 cells were cultured under low-glucose (LG; 5.6 mM D-glucose plus 27.4 mM D-mannitol) or high-glucose (HG; 33 mM D-glucose) conditions. For pharmacological PCSK9 inhibition, cells were pretreated with evolocumab (0.25 μM) for 1 h and subsequently exposed to HG for 48 h. For PCSK9 silencing, HK-2 cells were transfected with PCSK9-targeting siRNA (GAAUGACUUUUAUUGAGCU, 25 nM, Thermo Fisher Scientific) or control siRNA for 24 h, followed by exposure to HG for 48h. Protein levels of phospho-Thr^{172} AMPK, total AMPK, PGC-1α, and SREBP-1c were assessed by Western blotting as described in the Methods. Experiments were independently repeated at least three times. Statistical analysis was performed using one-way ANOVA followed by Dunnett's multiple-comparisons test.

Supplementary Figure S1. Effect of SBC-115076 on HK-2 cell viability. HK-2 cells were cultured under low-glucose (LG) or high-glucose (HG) conditions and treated with SBC-115076 (0–1 μM) for 48 h. MTT metabolic activity was expressed as a percentage of the LG vehicle control. Treatment with 0.25 μM SBC-115076 did not significantly affect MTT metabolic activity compared with the corresponding vehicle control under either LG or HG conditions. Data are presented as the mean ± SD from three independent experiments (n = 3). Statistical analysis was performed using two-way ANOVA followed by Dunnett's multiple-comparisons test. The adjusted p values for 0.1, 0.25, 0.5, and 1 μM SBC-115076 versus the corresponding vehicle control were 0.9925, 0.8874, 0.0004, and < 0.0001 under LG conditions and 0.6555, > 0.9999, 0.0004, and < 0.0001 under HG conditions, respectively. $^{***}p < 0.001$ vs. LG control; $^{†††}p < 0.001$ vs. HG control.

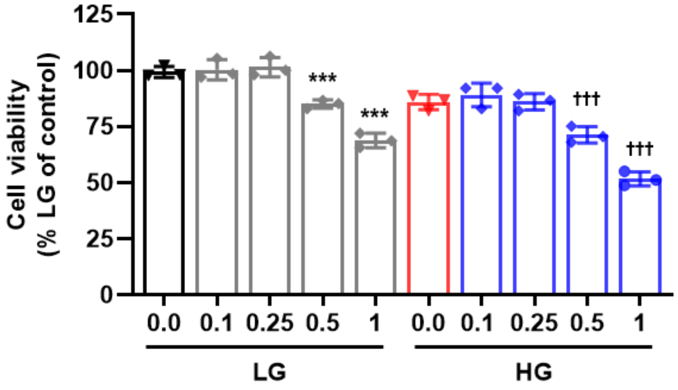

Supplementary Figure S2. Time-course validation of the efficiency and isoform selectivity of AMPKα1 and AMPKα2 knockdown in HK-2 cells. HK-2 cells were transfected with siRNA targeting PRKAA1 (A) or PRKAA2 (B) and harvested at 0, 24, and 48 h after transfection. Representative immunoblots and quantitative analyses of AMPKα1, AMPKα2, and total AMPK protein expression are shown. Protein expression was normalized to β-actin and expressed relative to the corresponding 0-h value. *PRKAA1* siRNA reduced AMPKα1 and total AMPK expression without significantly affecting AMPKα2 expression, whereas *PRKAA2* siRNA reduced AMPKα2 and total AMPK expression without significantly affecting AMPKα1 expression. Data are presented as the mean ± SD from three independent experiments (n = 3). Statistical analysis was performed using one-way ANOVA followed by Dunnett's multiple-comparisons test. * $p < 0.05$ and *** $p < 0.001$ vs. 0 h.

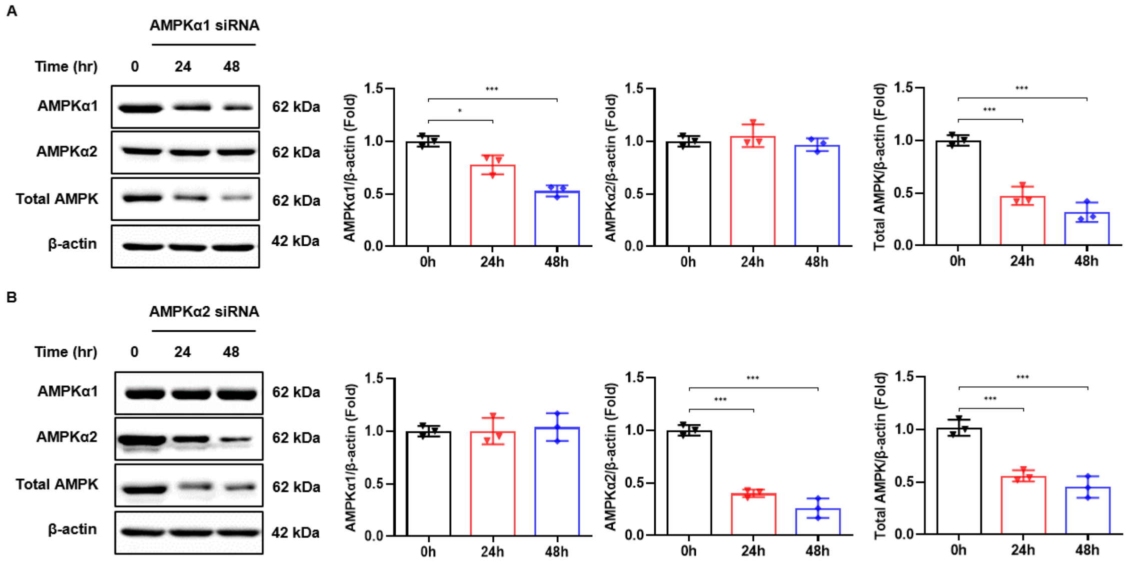

Supplementary Figure S3. Changes in histological findings of the liver in *db/db* mice treated with PCSK9 inhibitors. Representative liver sections stained with H&E are shown (original magnification, ×100). Steatosis was graded on a 0–3 scale based on the percentage of hepatocytes containing lipid droplets. Data are expressed as mean ± SD (n = 6 per group: *db/m* and *db/m* + PCSK9i, n = 8 per group *db/db* and *db/db* + PCSK9i). * $p < 0.05$, ** $p < 0.01$, and *** $p < 0.001$.

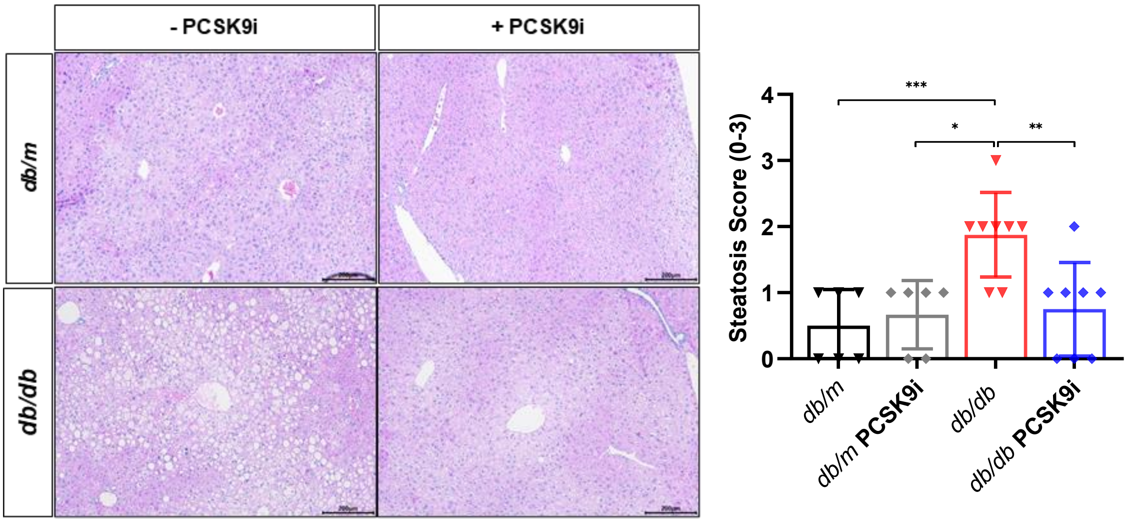

Supplementary Figure S4. Effects of evolocumab and PCSK9 silencing on AMPK-related signaling in high-glucose-treated HK-2 cells. (A) Representative immunoblots and quantitative analyses of phospho-Thr^{172} AMPK/AMPK, PGC-1α, and SREBP-1c in HK-2 cells exposed to low glucose (LG), high glucose (HG), or HG with evolocumab (0.25 μM). (B) Representative immunoblots and quantitative analyses of phospho-Thr^{172} AMPK/AMPK, PGC-1α, and SREBP-1c in HK-2 cells exposed to LG or HG after transfection with control siRNA, or to HG after transfection with PCSK9-targeting siRNA. Both evolocumab treatment and PCSK9 silencing increased the phospho-AMPK/AMPK ratio and PGC-1α expression and decreased SREBP-1c expression under HG conditions. Data are expressed as mean ± SD from three independent experiments (n = 3). * $p < 0.05$, ** $p < 0.01$, and *** $p < 0.001$.

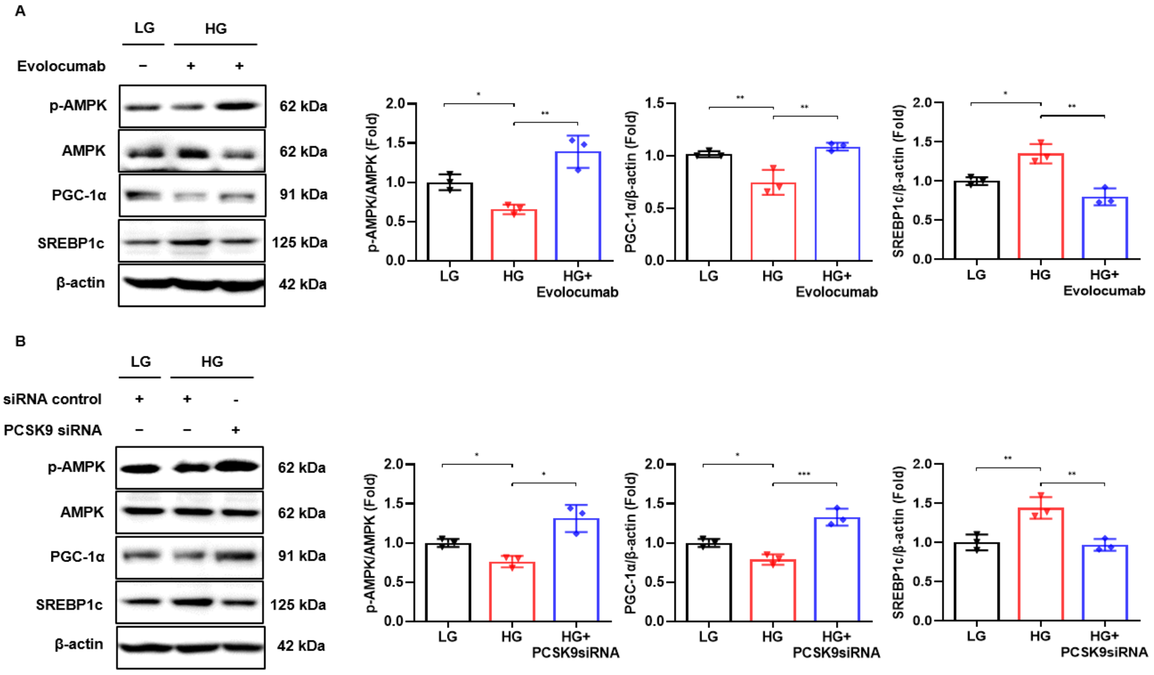

Supplementary Table S1. Commercial Assays and ELISA Kits

Assay	Sample	Supplier	Catalog No.
DCA HbA1c Reagent kit	Whole blood	Siemens Healthineers	10698915
Mouse Insulin ELISA kit	Serum	ALPCO	80-INSMS-E01
Mouse/Rat Cystatin C immunoassay kit	Serum	R&D Systems	MSCTC0
LDL-Cholesterol Assay Kit	Serum, Kidney	Crystal Chem Inc.	79980
LabAssay™ Non-Esterified Fatty Acid Assay Kits	Serum, Kidney	Wako	294-63601
Mouse Albumin ELISA Kit	Urine	Bethyl Laboratories	E99-134
Mouse TIM-1/KIM-1 ELISA Kit	Urine, Serum	R&D Systems	MKM100
Mouse Lipocalin-2/NGAL ELISA Kit	Urine, Serum	R&D Systems	MLCN20
ApopTag Peroxidase In situ Apoptosis Detection Kit	Kidney, HK-2 cells	Millipore	S7100
BODIPY staining	HK-2 cells	Thermo Fisher Scientific	D3821
H2DCFDA (H2-DCF, DCF)	HK-2 cells	Thermo Fisher Scientific	D399
Long-Chain Fatty Acid Oxidation Assay Kit	HK-2 cells	Assay Genie	BR00001-L
MDA assay kit	Kidney, HK-2 cells	Cell Biolabs Inc.	STA-832

Supplementary Table S2. Antibodies Used in Western Blotting and Immunohistochemistry

Target	Application	Dilution	Supplier	Catalog No.
PCSK9	WB	1:1000	HUABIO	EM1701-26
	IHC	1:100	Thermo Fisher Scientific	MA5-32843
LDLR	WB	1:2000	R&D Systems	AF2255
CD36	WB	1:1000	R&D Systems	NB400-144
Total AMPK	WB	1:1000	Cell Signaling Technology	2532
Phospho-AMPK (Thr^{172})	WB	1:500	Cell Signaling Technology	2535
AMPKα1	WB	1:1000	Cell Signaling Technology	2795
AMPKα2	WB	1:1000	Cell Signaling Technology	2757
PPARα	WB	1:1000	LSBio	LS-C344153
PPARγ	WB	1:1000	Thermo Fisher Scientific	MA5-32533
PGC-1α	WB	1:1000	Boster Biological Technology	M00236
Total Akt	WB	1:1000	Cell Signaling Technology	9272
Phospho-Akt (Ser^{473})	WB	1:1000	Cell Signaling Technology	9271
Total FoxO1	WB	1:500	Novus Biologicals	NBP2-31376
Phospho-FoxO1 (Ser^{256})	WB	1:1000	Novus Biologicals	NB100-81927
Total FoxO3a	WB	1:1000	Novus Biologicals	NBP2-16521
Phospho-FoxO3a (Ser^{253})	WB	1:2000	Novus Biologicals	NBP2-67521
SREBP1c	WB	1:1000	Novus Biologicals	NB600-582
SREBP2	WB	1:1000	Novus Biologicals	NB100-74543
Perilipin	WB	1:1000	ABclonal Korea	A4758
CPT1	WB	1:1000	Origene Technologies	TA332600
Bax	WB	1:1000	Abcam	ab182734
Bcl-2	WB	1:1000	Abcam	ab182858
GAPDH	WB	1:2000	Cell Signaling Technology	5174

β-actin	WB	1:20000	Sigma-Aldrich	A3854
TGF-β1	IHC	1:250	R&D Systems	MAB240
Type IV collagen	IHC	1:100	Novus Biologicals	NB120-6586
8-OHdG	IHC	1:200	JaICA	MOG-020P
Secondary Ab. (anti-rabbit HRP)	WB	1:20000	Cell Signaling Technology	7074
Secondary Ab. (anti-mouse HRP)	WB	1:20000	Cell Signaling Technology	7076

Supplementary Table S3. siRNA Sequences

Target	Gene symbol	Accession number	Sequence (5'→ 3')	Concentration	Supplier
AMPKα1	*PRKAA1*	NM_006251	GCAUAUGCUGCAGGUAGAU	25 nM	Bioneer
AMPKα2	*PRKAA2*	NM_006252	CGUCAUUGAUGAUGAGGCU	50 nM	Bioneer
PCSK9	PCSK9	NM_174936	GAAUGACUUUUAUUGAGCU	25 nM	Thermo Fisher Scientific

Supplementary Table S4. Primer Sequences for qRT-PCR

Gene	Species	Accession number	Forward Primer (5'→ 3')	Reverse Primer (5'→ 3')	Supplier
Pcsk9	Mouse	AY273821.1	GTCCTTCAGAGCAGGTCACTG	GACTCTTGCCACACACAGGTT	Genotech
Srebf1	Mouse	BC056922.1	AGCAGCCCCTAGAACAAACAC	CTTGATGAAGTGTGGCTGCAAT	Genotech
Perilipin	Mouse	AY035850.1	GTGTGTGAGATGGCCGAGAA	CTCCATCCTGTCTAGCCCCT	Genotech
Cpt1a	Mouse	BC054791.1	TTATCGTGGTGGTGGGTGTG	ACGCCACTCACGATGTTCTT	Genotech
Ppara	Mouse	AK035676.1	CACGATGCTGTCCTCCTTGA	GTCACAGAACGGCTTCCTCA	Genotech
Pparg	Mouse	AB256530.1	GCCCAGGTTTGCTGAATGTG	TGAGGACTCAGGGTGGTTCA	Genotech
Gapdh	Mouse	AK002273.1	TGCAGTGGCAAAGTGGAGATT	CGTGAGTGGAGTCATACTGGAACA	Genotech
